# Transcription factor E2F8 is a therapeutic target in the basal-like subtype of breast cancer

**DOI:** 10.3389/fonc.2023.1038787

**Published:** 2023-02-06

**Authors:** Jing Zheng, Jingyi Huang, Jinquan Xia, Wenbin Zhou, Lingyun Dai, Sihang Lin, Lin Gao, Chang Zou

**Affiliations:** ^1^ Department of Ultrasound, The Second Clinical Medical College, Jinan University (Shenzhen People’s Hospital), The First Affiliated Hospital of Southern University of Science and Technology, Shenzhen, Guangdong, China; ^2^ Department of Clinical Medical Research Center, The First Affiliated Hospital, School of Medicine, Southern University of Science and Technology, Shenzhen, China; ^3^ Department of Clinical Medical Research Center, The Second Clinical Medical College, Jinan University (Shenzhen People’s Hospital), The First Affiliated Hospital of Southern University of Science and Technology, Shenzhen, Guangdong, China; ^4^ Department of Thyroid and Breast Surgery, Department of General Surgery, The Second Clinical Medical College, Jinan University (Shenzhen People’s Hospital), The First Affiliated Hospital of Southern University of Science and Technology, Shenzhen, Guangdong, China; ^5^ Shenzhen Public Service Platform on Tumor Precision Medicine and Molecular Diagnosis, Shenzhen, Guangdong, China; ^6^ School of Life and Health Sciences, The Chinese University of Kong Hong, Shenzhen, Guangdong, China

**Keywords:** breast cancer, transcription factors, E2F8, biomarker, immune checkpoint molecules

## Abstract

**Introduction:**

Tumorigenesis in breast cancers usually accompanied by the dysregulation of transcription factors (TFs). Abnormal amplification of TFs leads aberrant expression of its downstream target genes. However, breast cancers are heterogeneous disease with different subtypes that have distinguished clinical behaviours, and the identification of prognostic TFs may enable to provide diagnosis and treatment of breast cancer based on subtypes, especially in Basal-like breast cancer.

**Methods:**

The RNA-sequencing was performed to screen differential TFs in breast cancer subtypes. The GEPIA dataset analysis was used to analyze the genes expression in invasive breast carcinoma. The expression of MYBL2, HOXC13, and E2F8 was verified by qRT-PCR assay in breast cancers. The depiction analysis of co-expressed proteins was revealed using the STRING datasets. The cellular infiltration level analysis by the TISIDB and TIMER databases. The transwell assay was performed to analyze cellular migration and invasion. CCK-8 assay was used to evaluate cellular drug susceptibility for docetaxel treatment. Predicted targeted drugs in breast cancers by GSCA Lite database online.

**Results:**

Kaplan-Meier plotter suggested that high expression of both E2F8 and MYBL2 in Basal-like subtype had a poor relapse-free survival. Functional enrichment results identified that apoptosis, cell cycle, and hormone ER pathway were represented the crucial regulation pathways by both E2F8 and MYBL2. In the meantime, database analysis indicated that high expression of E2F8 responded to chemotherapy, while those patients of high expression of MYBL2 responded to endocrinotherapy, and a positive correlation between the expression of E2F8 and PD-L1/CTLA4. Our cell line experiments confirmed the importance of E2F8 and MYBL2 in proliferation and chemotherapy sensitivity, possibly, the relationship with PD-L1. Additionally, we also observed that the up-regulation of E2F8 was accompanied with higher enrichments of CD4+ T cells and CD8+ T cells in breast cancers.

**Conclusion:**

Taken together, our findings elucidated a prospective target in Basal-like breast cancer, providing underlying molecular biomarkers for the development of breast cancer treatment.

## Introduction

Breast cancers are classified into four subtypes based on the traditional PAM50 algorithm, such as luminal A, luminal B, HER2-positive, and basal-like subtypes. The basal-like subtype accounts for 15%–20% of total breast cancers, and they have a higher risk of metastasis and early recurrence (1). Although chemotherapy is applied for basal-like subtypes, most of the patients still have not significantly benefited from it (2). The lack of available biomarkers for the basal-like subtype becomes an important challenge for treatment at present. Additionally, previous studies have also explored some targeted therapies for basal-like breast cancer, but the treatment efficacy of targets is limited (2). Hence, it is necessary to discover novel and available therapeutic targets for the basal-like subtype.

Transcription factors (TFs) directly regulate DNA through specialized DNA-binding motifs, including gene-specific promoters or enhancers (3). Breast cancers are characterized by the dysregulation and mutation of transcription factors such as ER-α, TP53, KLF5, SIX1, RUNX2, FOXO, MYC, and BRD4, which are capable of regulating epithelial-mesenchymal transition (EMT), the Warburg Effect, and mediating breast cancer cell resistance (4–9). Transcriptional regulators, the E2F family (E2F1-E2F8), contribute to regulating the cancer cell cycle, including *trans*-repression and *trans*-activation. There is a conserved DNA-binding domain in the *E2F8* gene to trans-repress its target gene promoters in cell development (10). E2F transcription factor 8 (E2F8) belongs to the atypical repressor family, which correlates with a higher proliferation of cells (11–13). Furthermore, *E2F7* and *E2F8* genes are targeted by E2F1, which results in the oscillation of the mRNA and protein of E2F7/8 throughout the late G1- and early S-phases of the cell cycle. Thus, the negative feedback between E2F7/8 and E2F1 maintains pro-proliferative and pro-apoptotic cellular activities in modulating cell cycle and cell death (13, 14). In breast cancer, the combination of E2F8 and hypoxia-inducible factor 1 (HIF1) has been shown to promote angiogenesis by inducing the expression of vascular endothelial growth factor A (VEGFA) (15). However, its functional and mechanical roles in basal-like breast cancer remain unclear. Moreover, the functional role of E2F8 in cancers is controversial. Several studies have revealed that E2F8 acts as a transcriptional activator, positively increasing the expression of cyclin D1 (CCND1) in tumor cells, and a high expression level of E2F8 has been linked with poor prognosis. However, other studies also identify E2F8 as a tumor suppressor (16, 17). Whether this difference is on account of the cellular heterogeneity or cancer cell stage (primary versus metastasis), it needs to be further explored.

MYB proto-oncogene like 2 (MYBL2), a Myb-related protein, is a proto-oncogenic transcription factor that contains a highly conserved helix-turn-helix DNA-binding domain. In breast cancers, MYBL2 can transactivate the *MYC* and *CCNB1* genes by binding their promoters and expediting the cell cycle of cancer cells (18). Moreover, MYBL2 also involves itself in promoting cell stemness by controlling targets such as *NANOG*, *KLF4*, *SND1*, and *JUN* (19). Rising evidence has suggested that MYBL2 is frequently overexpressed in breast cancer and other solid tumors, and it has a positive correlation with the poor prognosis of patients with solid tumor. Increasing MYBL2 promotes cell cycle progression and inhibits cell apoptosis through several pathways; for example, it accelerates the cell cycle in S-phase by combining partner *TAF15* and *MuvB* genes to transcriptionally activate RRM2, which enhances the malignancy of colorectal cancer cells (20). Additionally, MYBL2 can be dephosphorylated by activated PPP2R5E(B56ϵ)/iHAP1 complexes, causing tumor cells to enter an irreversible prometaphase (21). However, the exact function of MYBL2 in basal-like breast cancer needs to be clarified. Furthermore, the high expression level of MYBL2, E2F1, and FOXM1 is positively associated with TP53 mutations in breast cancer progression (22).

In this study, we found that both E2F8 and MYBL2 were essential transcription factors in the basal-like subtype, especially E2F8. Upregulated expressions of both E2F8 and MYBL2 were associated with poor prognosis in the basal-like subtype. The cell cycle was significantly accelerated, and the hormone estrogen receptor pathway was repressed by E2F8 in breast cancers. The patients with upregulated E2F8 had a better response to chemotherapy. Furthermore, our data also showed that the upregulation of E2F8 was linked to higher enrichments of CD4+ and CD8+ T cells in breast cancers. The results showed a positive correlation between the expression of E2F8 and immune checkpoint inhibitors. Based on these findings, we hypothesized that E2F8 could be used as an underlying target in basal-like breast cancer treatment.

## Materials and methods

### Total RNA extraction and high-throughput next-generation RNA sequencing

Total RNA was isolated from human breast cancer tissues and adjacent tissues using the TRIzol reagent. The RNA concentration was determined by the Qubit system, purified RNA was assessed by the Nanodrop system (OD260/280), and the integrality of RNA was detected using the Agilent 2100 system. RNA sequencing was performed on the Illumina Novaseq 6000 platform.

### The differential expression analysis in breast cancer subtypes

All breast cancer tumors and paired adjacent tissues were removed during surgical operations that were diagnosed between 2018 and 2021 at the Department of Thyroid and Breast Surgery, Shenzhen People’s Hospital (including three cases of luminal A, three cases of luminal B, three cases of HER2 positive, and three cases of basal-like subtype). The breast cancer datasets were obtained from The Cancer Genome Atlas Data (TCGA) database (http://cancergenome.nih.gov/). The clinical subtypes of breast cancer were analyzed based on PAM50 classification by R software (23). The differential gene expression was performed by edgeR analysis (24). Both a *p*-value of <0.05 and a threshold log_2_[fold change] of ≥3 were used to evaluate the significant difference in the data.

### GEPIA (GEPIA 2) datasets and Kaplan–Meier plotter

The Gene Expression Profiling Interactive Analysis (GEPIA) (https://gepia.cancer-pku.cn/) was used to verify the differentially expressed genes between breast cancers and paracancerous tissues. GEPIA was a newly developed tool for analyzing genetic differences based on TCGA datasets. The GEPIA 2 dataset (http://gepia2.cancer-pku.cn/#index) was the updated version of the GEPIA database that was used to identify the expression levels of both E2F8 and MYBL2. The Kaplan–Meier plotter (https://www.kmplot.com) online was performed to analyze and demonstrate the prognostic survival curves of breast cancer patients. The survival curves of patients were plotted in high gene expression and low expression groups in terms of both hazard ratios with 95% confidence intervals and a *p*-value of <0.05.

### Quantitative reverse transcription-polymerase chain reaction

The fresh breast cancer tissues were ground after using a liquid nitrogen flash freezer. Total RNA was isolated and extracted using a TRIzol reagent. The transcript One-Step RT-PCR SuperMix (Transgen Biotech) kit was used to perform reverse transcription reactions according to the manufacturer’s instructions. cDNAs were amplified by SYBR Green PCR Master Mix (Thermo Fisher Scientific). The quantitative reverse transcription-polymerase chain reaction (qRT-PCR) primers were generated by Integrated DNA Technologies (https://sg.idtdna.com/pages) online database, and the producing primers were BLAST using the NCBI (https://www.ncbi.nlm.nih.gov/) online database. All primer sequences are listed in **Supplementary Table 5**. The expression levels of mRNA were calculated using the comparative cycle threshold values. Three biological replicates were performed for each experiment.

### Gene set cancer analysis (GSCALite) database

GSCALite (http://bioinfo.life.hust.edu.cn/web/GSCALite/) is a web server for analyzing a series of genes related to cancers online (25). The differential gene expression, genomic variations, and associated signaling pathways are shown and evaluated using the GSCALite database.

### Protein–protein interaction network and GO analysis

The protein–protein interaction (PPI) network constituted the associations of cellular function (26). The PPT network information for TFs was obtained from the STRING database (https://cn.string-db.org/), and the PPI image was depicted using Cytoscape (3.7.2) software. Gene Ontology (GO) analysis was used to analyze the molecular functions of TFs.

### Tumor immune estimation resource database and TISIDB database

The tumor immune estimation resource (TIMER; https://cistrome.shinyapps.io/timer/) dataset was performed to explore the relationship between the expression levels of E2F8 (MYBL2) and the immune cell infiltration levels based on copy number alteration of somatic E2F8 (MYBL2). An integrated repository portal for tumor-immune system interactions (TISIDB) database was used to analyze the correlation between the expression levels of E2F8 (MYBL2) and the immune cellular subtypes (http://cis.hku.hk/TISIDB/).

### Statistics analysis

All statistical data were analyzed using the SPSS 21.0 software (IBM Corp., Armonk, NY, USA). Differential gene expression curves were drawn by GraphPad Prism Software 7.0 (GraphPad Inc, La Jolla, CA, USA). QRT-PCR results were analyzed as the mean ± standard deviation (SD). Relapse-free survival and overall survival curves were analyzed and drawn by the log-rank test. ROC analysis for sensitivity (%) and specificity (%) of patients was presented by the area under the curve (AUC). The correlation analysis was shown by Pearson’s test. All data were calculated by a normal distribution and homogeneity of variance. The means were compared using independent-sample *t*-tests for two groups or one-way analysis of variance (ANOVA) for more than three groups. LSD analysis was adopted for equal variance, while Dunnett’s T3 analysis was used for unequal variance. A *p*-value cutoff was shown using ^*^ *p* < 0.05, ^**^ *p* < 0.01, and ^***^p < 0.001, respectively.

## Results

### Identifying differential expression of TFs in breast cancer subtypes

**Figure 1:**
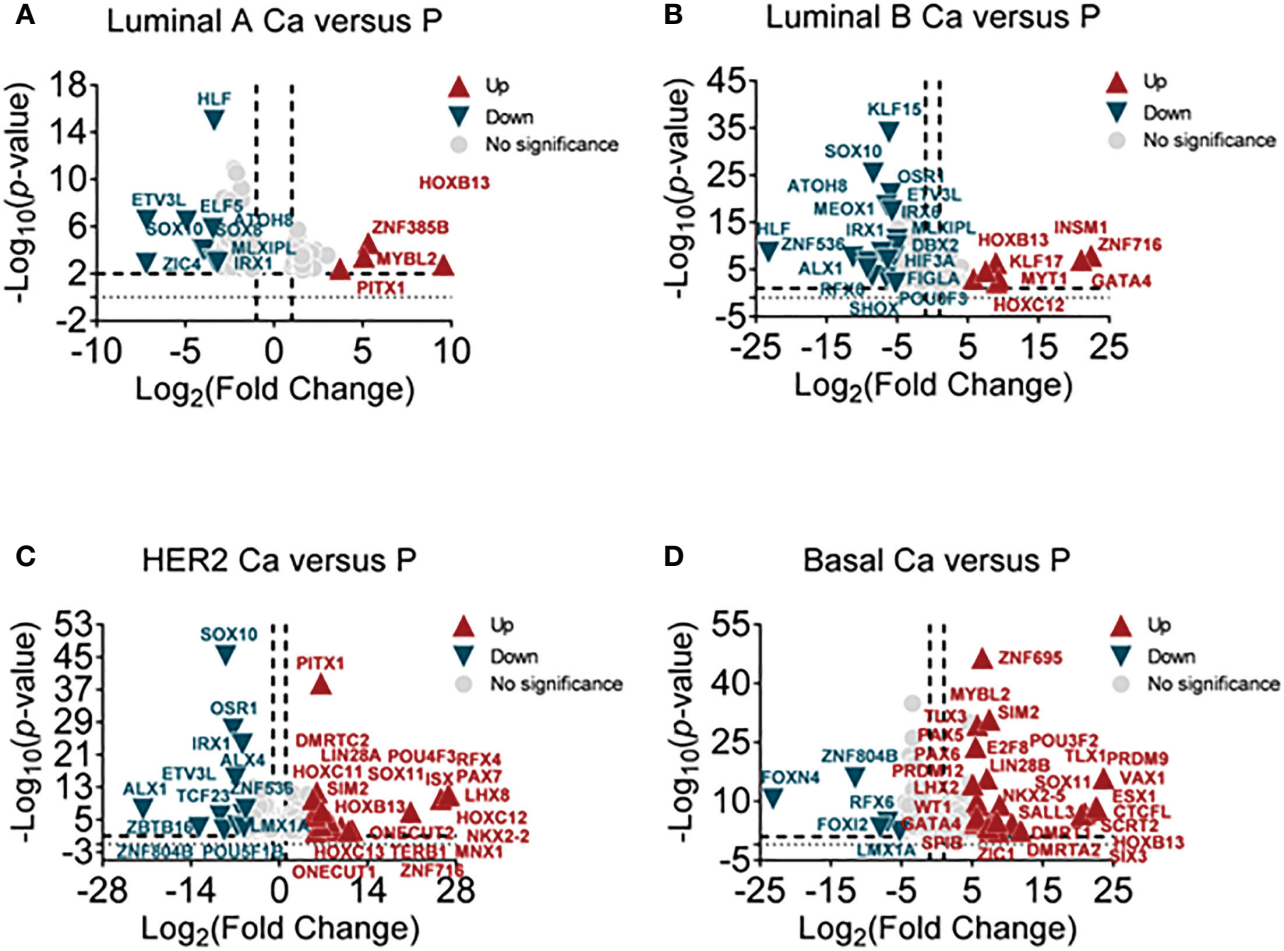
Screening differential TFs in breast cancer subtypes by RNA-sequencing. **(A)** The volcano plot analysis of high and low TFs in the luminal A subtype (luminal A Ca) compared with adjacent tissue (luminal A P) (*n* = 3), **(B)** in luminal B subtype (luminal B Ca) compared with adjacent tissue (luminal B P) (*n* = 3), **(C)** in the HER2 subtype (HER2 Ca) compared with adjacent tissue (HER2 P) (*n* = 3), and **(D)** in basal-like subtype (basal Ca) compared with adjacent tissue (basal P) (*n* = 3).

According to clinical histopathologic diagnosis, 12 cases of breast cancers had been classified as four subtypes, in which the cases had matched adjacent tissues. A high-throughput RNA sequence was performed to identify differential transcription factors in these samples. These subtypes were classified as follows (1): luminal A (estrogen receptor (ER)+/progestogen receptor (PR)+/HER2 receptor−) (2); luminal B (ER+/PR+/HER2+) (3); human epithelial factor receptor (HER)2 positive (ER−/PR−/HER2+); and (4) basal-like (75% belong to triple-negative breast cancer, ER-/PR-/HER2-). The NOI-seq algorithm (27) was performed to filter four upregulated and nine downregulated TFs in luminal A cancers compared with normal tissues (**Figure 1A**, **Supplementary Table 1**). It showed seven upregulated and 18 downregulated TFs in luminal B cancers compared with normal tissues (**Figure 1B**, **Supplementary Table 2**). It identified 20 upregulated and 12 downregulated TFs in HER2 cancers compared with normal tissues (**Figure 1C**, **Supplementary Table 3**). In addition, it verified 28 upregulated and five downregulated TFs in basal-like cancers compared with normal tissues (**Figure 1D**, **Supplementary Table 4**). These data suggested that it could provide infusive molecular targets for different subtypes of breast cancers.

### The correlation between MYBL2, HOXC13, E2F8, and clinical prognosis of breast cancer patients

**Figure 2:**
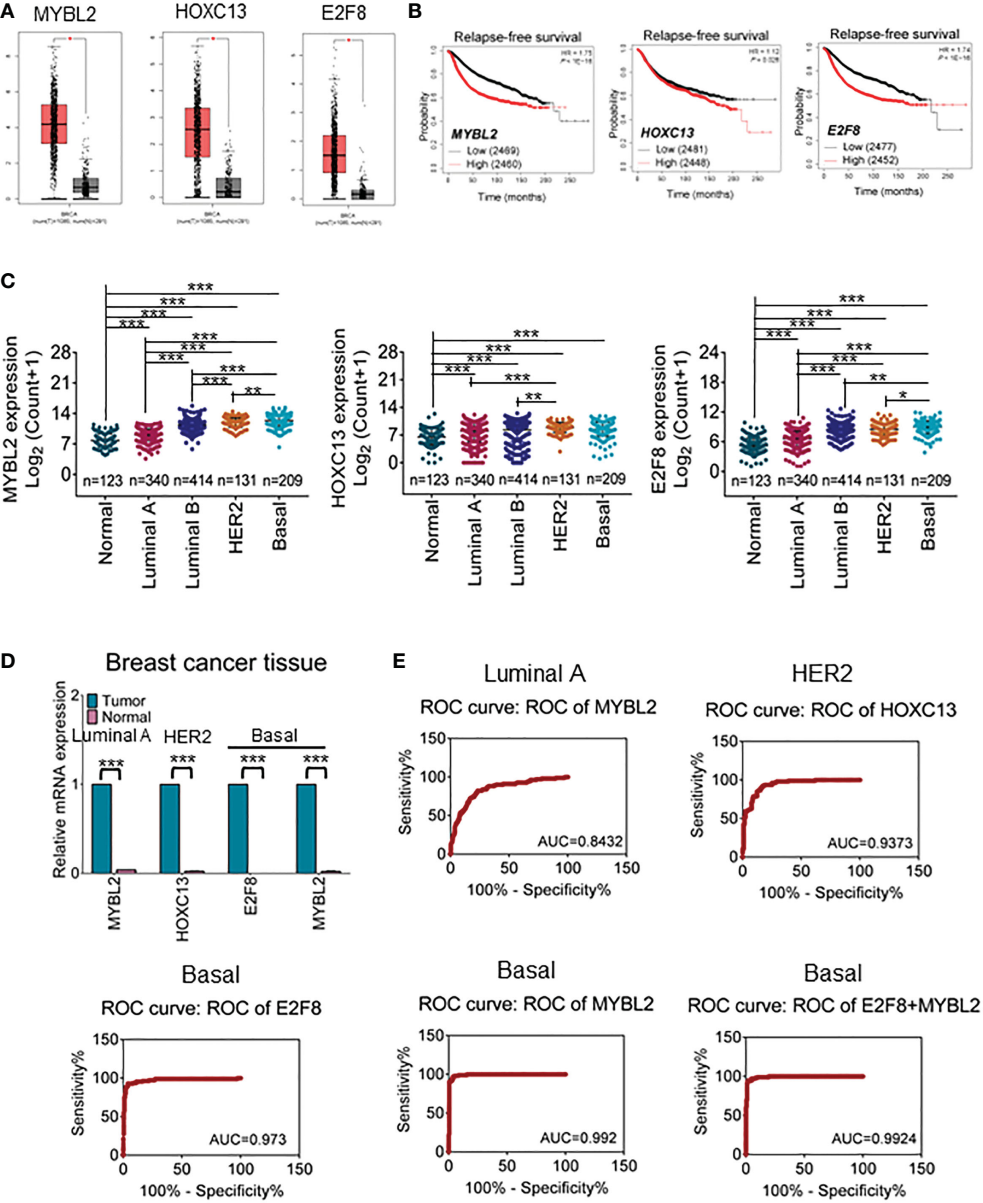
The prognosis value for upregulation of TFs in breast cancers. **(A)** The GEPIA dataset analysis for the expression of MYBL2, HOXC13, and E2F8 in invasive breast carcinoma. **(B)** The Kaplan–Meier plotter dataset analysis for relapse-free survival (RFS) of patients who had “high” or “low” expression of E2F8. **(C)** The expression analysis of MYBL2, HOXC13, and E2F8 in breast cancer subtypes from TCGA data based on PAM50 classification. **(D)** The expression of MYBL2, HOXC13, and E2F8 was verified by qRT-PCR assay in breast cancers. **(E)** Illustrative ROC curves for MYBL2, HOXC13, and E2F8 from TCGA data based on PAM50 classification of breast cancers (^*^ *p* < 0.05; ^**^ *p* < 0.01; ^***^ *p* < 0.001).

Abundant evidence revealed that TFs are differentially expressed in cancers and contributed to regulating cancer stem cells, invasion, metastasis, and cellular metabolism (28). To further narrow the available biomarkers, we validated the mRNA expression levels of filtered TFs in large samples of breast cancers using the GEPIA online database. The results showed that HOXC13 had a high expression in HER2 cancers and E2F8 had a high expression in basal-like cancers compared with normal tissues (**Figure 2A**). Furthermore, the Kaplan–Meier dataset plotter was performed to reveal that high expression of MYBL2, HOXC13, and E2F8 had a poor RFS rate in invasive breast cancers (**Figure 2B**). Additionally, based on canonical PAM50 classification, we downloaded TCGA data for four breast cancer subtypes. These results showed that the expression of MYBL2 was upregulated in the basal-like subtype compared to other subtypes and normal tissue using TCGA data. The E2F8 had a high expression in the basal-like subtype compared to luminal A and luminal B subtypes but had no significance compared to the HER2 subtype. In addition, the HOXC13 had a high expression in the HER2 subtype compared to luminal A and luminal B subtypes but had no significance compared to the basal-like subtype (**Figure 2C**). Next, the qRT-PCR assay identified that E2F8 was upregulated in the basal-like subtype and HOXC13 was upregulated in the HER2-positive subtype. Interestingly, MYBL2 had higher expression levels in both luminal A and basal-like breast cancer tissues than in adjacent tissues (**Figure 2D**). Moreover, we also explored these TF expression levels in pan-cancers. The results showed that the expression levels of both E2F8 and MYBL2 were upregulated in female cancers compared to adjacent tissues, such as breast cancer (BRCA), ovarian cancer (OV), and cervical squamous cell carcinoma and endocervical adenocarcinoma (CESC). Moreover, they had high expression levels in lung adenocarcinoma (LUAC) and lung squamous cell carcinoma (LUSC) (**Supplementary Figure 1**).

Next, to explore whether these TFs could become potential clinical biomarkers, ROC curves were performed to visualize the tradeoff between sensitivity and specificity in the clinic for each cutoff. Based on TCGA data, 326 cases of luminal A subtype, 131 cases of HER2-positive, 208 cases of basal-like subtype, and 115 cases of adjacent tissues were used for ROC analysis. The results had shown that the AUC of HOXC13 in the HER2-positive subtype was 0.9373 (95% confidence interval (CI): 90.85%–96.61%), the sensitivity was 96.46% (95% CI: 91.18%–99.03%), and the specificity was 74.05% (95% CI: 65.66%–81.31%). The AUC of E2F8 in the basal-like subtype was 0.973 (95% CI: 95.25%–99.36%), the sensitivity was 99.12% (95% CI: 95.17%–99.98%), and the specificity was 67.48% (95% CI: 60.62%–73.82%). The AUCs of MYBL2 were 0.842 (95% CI: 80.02%–88.62%) in luminal A and 0.992 (95% CI: 98.37%–100.00%) in the basal-like subtype; the sensitivity was 90.27% (95% CI: 83.25%–95.04%) in luminal A and 100.00% (95% CI: 96.79%–100.00%) in the basal-like subtype, while the specificity was 58.59% (95% CI: 53.03%–63.99%) in luminal A and 75.73% (95% CI: 69.28%–81.42%) in the basal-like subtype, respectively (**Figure 2E**). These results suggested that MYBL2 and E2F8 had considerable diagnostic values in the basal-like subtype.

### The regulatory signaling pathways and functions of E2F8

**Figure 3:**
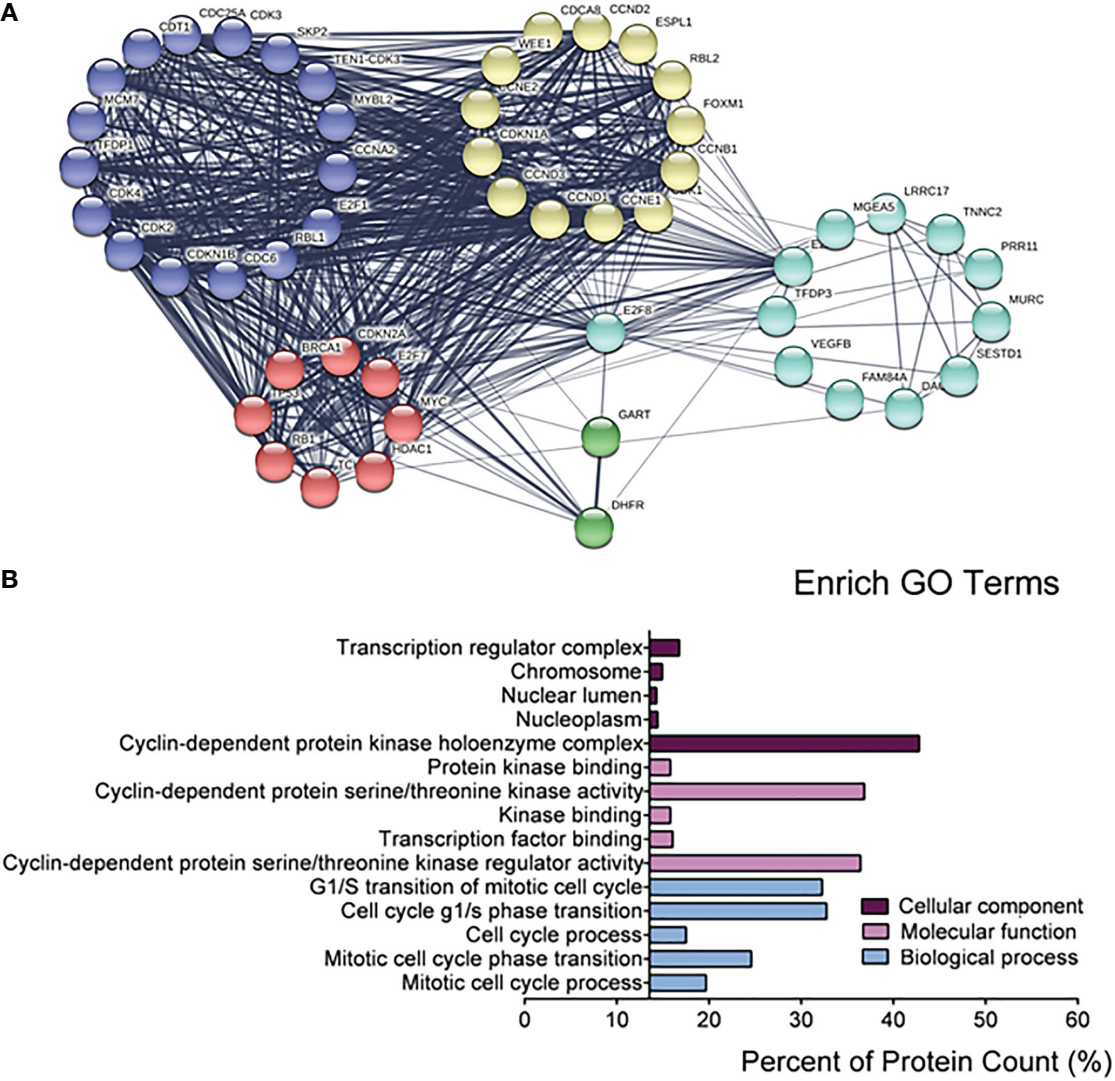
The regulatory networks for E2F8. **(A)** The depiction analysis between E2F8 and coexpressed proteins using the STRING datasets. **(B)** The converged GO analysis for E2F8 and its coexpressed proteins, including cellular components, molecular function, and biological process, using the GraphPad Prism software.

Because of the specificity of E2F8 in basal-like breast cancer, in further studying the regulation of pathway networks by E2F8, we analyzed the signaling pathway networks associated with E2F8 using the GSCALite online tool and the STRING database. The results had shown that E2F8 was able to promote the cell cycle process, but it could inhibit the hormone ER pathway in all breast cancers using the GSCALite dataset (**Supplementary Figure 2**). In addition, the expression of E2F8 was not only increased in the basal-like subtype but also in other subtypes in comparison with normal tissues using the TIMER dataset online (**Supplementary Figure 3A**). Similar results were observed in breast cancers with high expression of MYBL2 (**Supplementary Figure 3B**). Furthermore, the PPI interaction network showed 49 coexpressed proteins with E2F8, most of which were cancer-associated proteins. These coexpressed proteins of five clusters were listed, including transcription factors (MYC, E2F7, BRCA1, TP53, RB1, HDAC1, etc.), cell cycle genes (CCND1, CCNE1, CCNB1, CCNE2, CCND3, etc.), and cyclin-dependent protein kinase activity (CDK3, CDK4, CDK2, CDKN1B, etc.). Interestingly, MYBL2 also interacted with E2F8 in the PPI networks (**Figure 3A**). Among the top 5 converged GO enrichment results, the data revealed that E2F8 mostly regulated mitotic processes, such as the cell cycle process and transcriptional regulation (**Figure 3B**). These findings implicated that E2F8 was correlated with breast progression and high expression of E2F8 could become a potential biomarker in basal-like breast cancer.

### The correlation between the expression of E2F8 and immune infiltration in breast cancer

**Figure 4:**
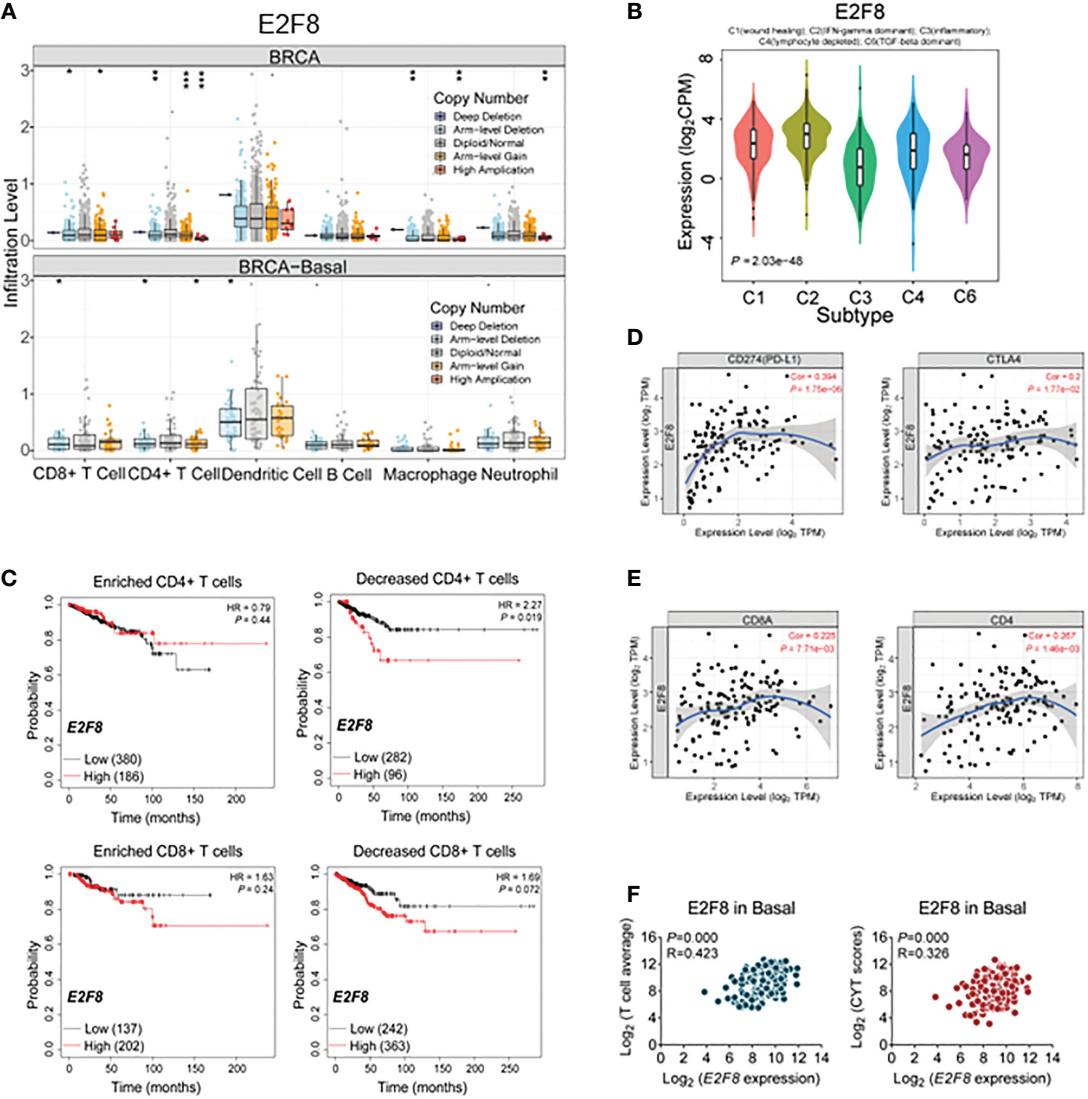
The immune cellular infiltrate analysis in breast cancers. **(A)** The cellular infiltration-level analysis in high expression of E2F8 in basal-like subtypes and breast cancer (BRCA) by the TISIDB database. **(B)** The correlation between E2F8 expression and immune subtype analysis in invasive breast cancers. The classic classification is as follows: C1 (wound healing), C2 (IFN-γ dominant), C3 (inflammatory), C4 (lymphocyte depleted), C5 (immunologically quiet), and C6 (TGF-β dominant). **(C)** The overall survival **(OS)** analysis of patients suffering from invasive breast cancer by Kaplan–Meier plotters. The OS groupings covered enriched or decreased immune cells in the patients who had “high” or “low” expression of E2F8. **(D)** The correlation between E2F8 expression and immune checkpoint molecules (PD-L1 and CTLA4). **(E)** The correlation between E2F8 expression and CD8A/CD4. **(F)** The correlation between E2F8 expression and T-cell average/CYT scores.

A multitude of evidence suggests that tumor infiltration lymphocytes (TILs) are involved in various cancer progressions (29). To explore a linker between the expression of E2F8 and immune cellular infiltration, we found that the expression level of E2F8 was observably associated with immune infiltration of the basal-like subtype and that most of the infiltrated immune cells were CD8+ T cells, CD4+ T cells, and dendritic cells. Additionally, the immune infiltration was also was affected by the variation or status of gene copy numbers using the TISIDB database analysis (**Figure 4A**). Likewise, the patients in the basal-like subtype who had upregulation of MYBL2 were accompanied by infiltration of CD8+ and CD4+ T cells (**Supplementary Figure 3A**). Similar results were found in patients with high expression of MYBL2 (**Supplementary Figure 4A**).

Next, the immune expression signatures between E2F8 and breast cancer were identified by TISIDB online (30). The six clusters of immune subtypes, C1–C4 and C6 (with C1 369, C2 390, C3 191, C4 92, and C6 40 cases for both E2F8 and MYBL2 genes, respectively), were used to characterize the signatures. We found that the expression level of E2F8 had an observable correlation with immune subtypes and that C2 (IFN-gamma dominant) was increased at the high expression level of E2F8 (**Figure 4B**). It had come up with similar results in high MYBL2 expression groups (**Supplementary Figure 4B**). Further analysis for the correlation between the expression level of E2F8 and immune cell subtypes, including those in breast cancers, revealed that the expression level of E2F8 was markedly positively related to these immune cells, especially CD8+ T cells, CD4+ T cells, neutrophil, and dendritic cells in the basal-like subtype, while the correlation between the expression level of MYBL2 and immune cells was not obviously exhibited in the basal-like subtype (**Supplementary Figure 5**). Moreover, the Kaplan–Meier plotter data revealed that the patients with both high E2F8 mRNA levels and decreased T cells (CD4+ T cells, CD8+ T cells, and macrophage cells) had a worse RFS compared with the patients with enriched CD4+ T cells in breast invasive carcinoma (**Figure 4C**). Similar results were shown in patients with high mRNA levels of MYBL2 (**Supplementary Figure 4C**). A previous study identified that breast cancer patients with high CD8+ T-cell infiltration, especially the basal-like subtype, had higher potential benefits from immune checkpoint molecules (ICMs), such as PD-1 and CTLA4 (31). We identified the correlation between E2F8 and 14 ICMs, also including CD4 and CD8. These data suggested that there were observably positive correlations between E2F8 and all the ICMs (including CD4 and CD8) (**Figure 4E**, **Supplementary Figure 6A**). We found a positive correlation not only between E2F8 and PD-L1/CTLA4 expression (Cor = 0.394) but also between E2F8 and CD8A/CD4 expression (Cor = 0.2) in basal-like breast cancer (**Figure 4D**), while MYBL2 had no significant correlations with ICI expression in basal-like breast cancer (**Supplementary Figures S4D, E, S6B**).

Moreover, we also found that in T-cell average gene signature (32), the expression of E2F8 was highly positively correlated with all of the T-cell infiltration-associated genes (*CCL2*, *CCL3*, *CCL4*, *CXCL9*, *CXCL10*, *CD8A*, *HLA-DOB*, *HLA-DMB*, *HLA-DO1*, *GZMK*, *ICOS*, and *IRF1*) in the basal-like subtype (*p* = 0.000; *R* = 0.423). A similar result was observed in the immune cytolytic activity (CYT) score (*GZMA* and *PRF1*) in basal-like breast cancer (*p* = 0.000; *R* = 0.326) (**Figure 4F**). These results showed that the patients with high expression of E2F8 could benefit from ICI treatment rather than MYBL2. Furthermore, increased E2F8 and CD+8 T cells could be incorporated as a biomarker into ICI-based patient management.

### Loss of E2F8 inhibits the cellular migration and invasion in basal-like breast cancer

**Figure 5:**
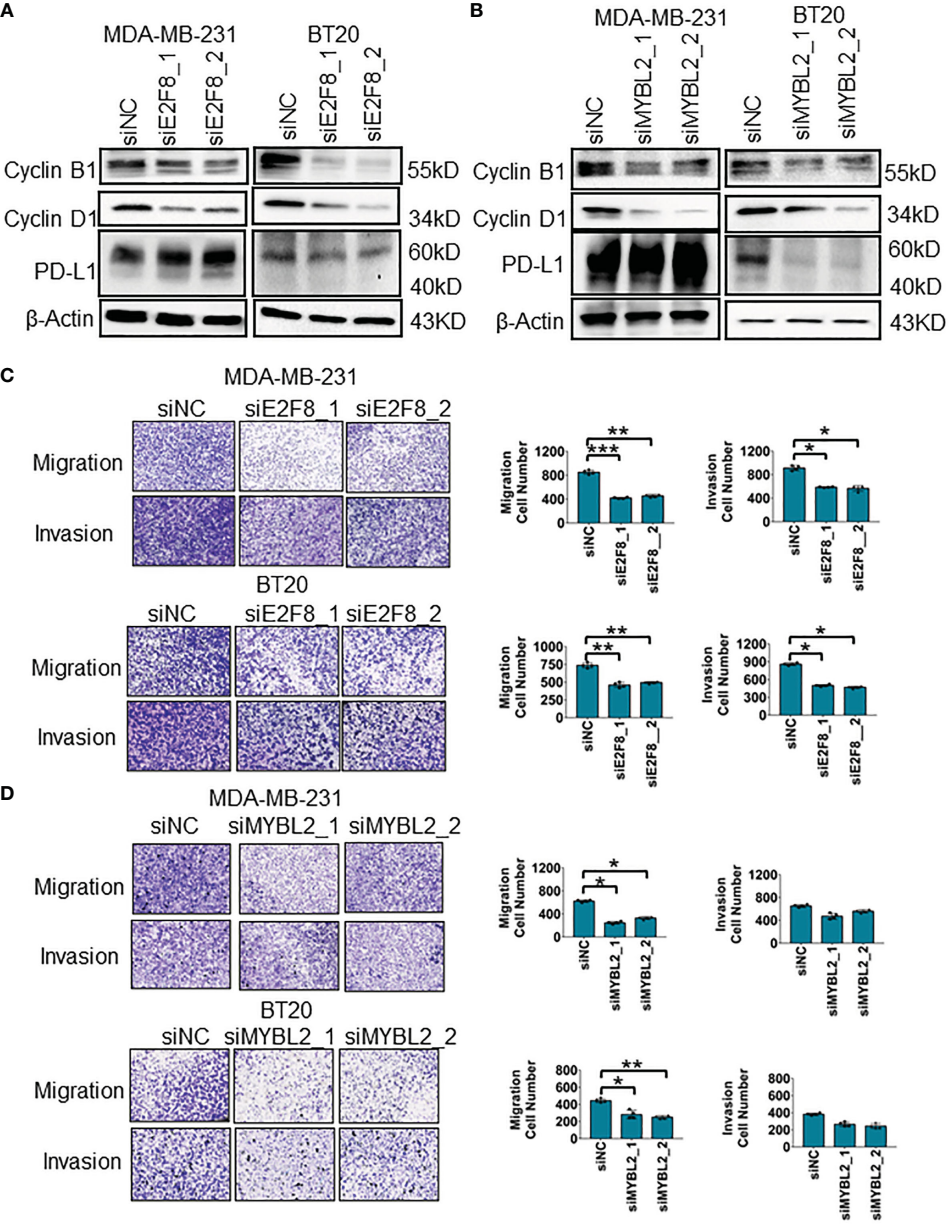
The migration and invasion analysis for basal-like breast cancer cells. **(A)** The expression of cyclin B1, cyclin D1, and PD-L1 in MDA-MB-231 and BT20 cells treated with E2F8 siRNA compared with siNC, as assessed by Western blot assay. **(B)** The expression of cyclin B1, cyclin D1, and PD-L1 in MDA-MB-231 and BT20 cells treated with MYBL2 siRNA compared with that of siNC by Western blot assay. **(C)** Transwell assay analysis for the migration and invasion of MDA-MB-231 and BT20 cells treated with siNC and siE2F8 oligos. **(D)** Transwell assay analysis for the migration and invasion of MDA-MB-231 and BT20 cells treated with siNC and siMYBL2 oligos. ^*^ *p* < 0.05; ^**^ *p* < 0.01; ^***^ *p* < 0.001 compared with the siNC group.

To uncover the oncogenic feature of E2F8 in basal-like breast cancer, we knocked down E2F8 expression using small interfering ribonucleic acid (siRNA) in MDA-MB-231 and BT20 cell lines. The expression level of E2F8 was downregulated significantly in cells treated with E2F8 siRNA oligos (siE2F8_1 or siE2F8_2) compared to negative control siRNA (siNC) in both cell lines (**Supplementary Figure 7A**). Furthermore, loss of E2F8 markedly inhibited the expression levels of cyclin B1 and cyclin D1 in both cell lines (**Figure 5A**). Similarly, the loss of MYBL2 also repressed the expression of cyclin B1 and cyclin D1 in both cell lines (**Figure 5B**). Interestingly, the loss of either E2F8 or MYBL2 inhibited the expression of PD-L1 in BT20 cells, but no significant change in MDA-MB-231 cells. Moreover, the knockdown of E2F8 inhibited cell migration and invasion in both cell lines (**Figure 5C**). Knockdown of MYBL2 could repress cellular migration in both cell lines but had no significant difference in cellular invasion compared to siNC groups in both cell lines (**Figure 5D**). These results suggested that a deficiency of E2F8 could repress the progression of basal-like breast cancer compared with MYBL2.

### Predicted therapeutic effect for patients with high expression of E2F8 in breast cancer

**Figure 6:**
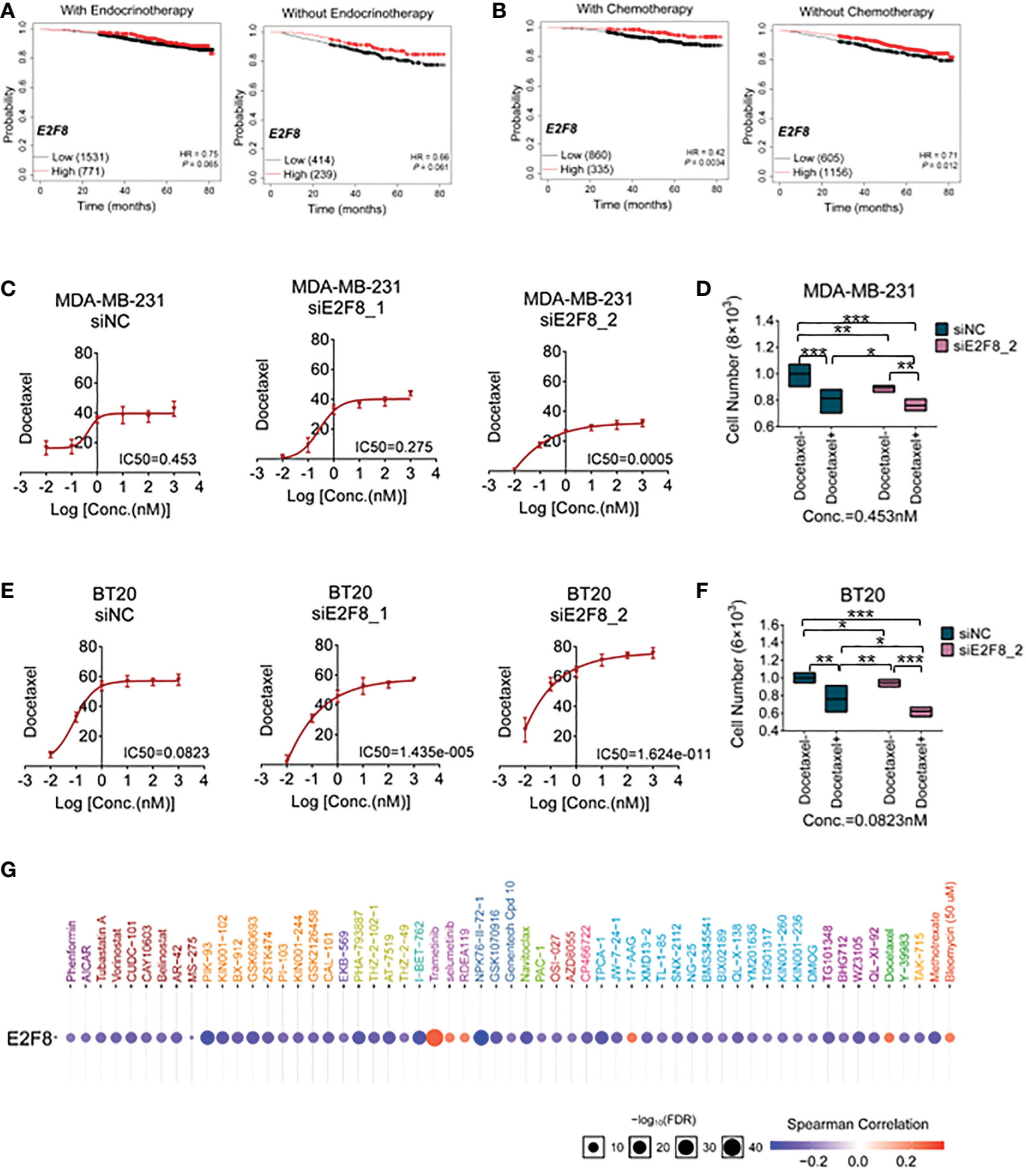
The treatment for breast cancer patients with high expression of E2F8. **(A)** Kaplan–Meier survival analyses of the overall survival **(OS)** of patients suffering from invasive breast cancer. The OS groupings covered patients “with” or “without” endocrinotherapy who had “high” or “low” expression of E2F8. **(B)** The OS groupings covered patients “with” or “without” chemotherapy who had “high” or “low” expression of E2F8 or MYBL2. CCK-8 analysis of the IC_50_ of cells treated with docetaxel for the siNC group, siE2F8 1 group, and siE2F8 2 group in **(C)** MDA-MB-231 cell and **(E)** BT20 cell lines. CCK-8 analysis of the drug susceptibility for the siNC group, siNC with the docetaxel-treated group, the siE2F8 group, and siE2F8 with the docetaxel-treated group in **(D)** MDA-MB-231 and **(F)** BT20 cell lines. **(G)** Targeted drugs in anti-E2F8 in breast cancers by the GSCALite online database. The sensitive drugs were represented by the blue color, while the resistant drugs were represented by the red color. *P<0.05, **P<0.01 and ***P<0.001 compared with control group.

To enhance basal-like subtype-based precision treatment, we analyzed the overall survival of patients with a high expression level of E2F8 treated with endocrinotherapy and/or chemotherapy. Compared to those patients without endocrinotherapy, the patients with endocrinotherapy had not got a better prognosis in the high expression level of the E2F8 group (**Figure 6A**). However, in comparison to endocrinotherapy, the patients who had high expression levels of E2F8 were more likely to benefit from chemotherapy (**Figure 6B**).

To explore the correlation between E2F8/MYBL2 and chemotherapy, docetaxel, a clinical first-line drug of chemotherapy, was used to test the sensitivity of breast cancer cells. Firstly, we detected the IC_50_ of docetaxel treatment for MDA-MB-231 and BT20 cell lines after the knockdown of E2F8. These results demonstrated that the IC_50_s of both MDA-MB-231 and BT20 cells that were treated with siE2F8 oligos were significantly decreased compared to the siNC group after docetaxel treatment (**Figures 6C, E**). However, it had no remarkable differences in siMYBL2-treated cell groups in both MDA-MB-231 and BT20 cells (**Supplementary Figures S8A, B**). Secondly, the CCK-8 assay showed that cell proliferation in the docetaxel-treated group was inhibited compared to the docetaxel-treated group. In both cell lines, cell numbers were notably decreased in cells treated with docetaxel and siE2F8 oligo-treated compared to those not treated with docetaxel and siNC (**Figures 6D, F**). These results suggested that the knockdown of E2F8 could enhance the sensitivity of docetaxel therapy and inhibit cell proliferation. In addition, there was no significant decrease between docetaxel and siMYBL2-treated cells and docetaxel and siNC-treated cells (**Supplementary Figures S8C, D**.). These data indicated that E2F8, which acted as a novel basal-like biomarker, had ascendancy in contrast to MYBL2.

Next, we predicted the potential efficacy of anti-E2F8-related targets through the GSCALite online database. Most drugs targeting E2F8 contained modules that were enriched in cell cycle and cellular proliferation processes. Based on screening these usable drugs, which had both minor *p*-values and better drug susceptibilities, we found that these drugs, such as PIK-93, I-BET-762, NPK76-II-72-1, and TPCA-1, could benefit patients of the basal-like subtype. However, other targeted drugs, such as trametinib, selumetinib, RDEA119, 17-AAG, docetaxel, and bleomycin (50 μM), may have developed resistance in patients with high expression of E2F8 (**Figure 6G**). These results showed that the patients who suffered from high expression levels of E2F8 had a discrepant prognosis after endocrinotherapy and chemotherapy for breast cancers. Patients with high E2F8 expression could benefit from novel targeted treatment.

## Discussion

Although there have been significant advances in targeted therapy for breast cancer in the past decades, the invasiveness and metastasis of breast cancer remain as major challenges for treatment (33). Breast cancers have been viewed as a highly heterogeneous population of cells, especially in the basal-like subtype (2). The TFs play crucial roles in regulating specific transcription of targets in breast cancers. The TF dysfunction and dysregulation can result in generating more cancerous cells. Our previous study also probed that some TFs, including Yin Yang 1 (YY1), as a molecular target for basal-like breast cancer, could promote epithelial-to-mesenchymal transition progression of the basal-like subtype through *trans*-activating the *Kinectin 1* gene (34). In addition, we also found that subunit p65 of NF-kappa(κ)B (NF-κB/p65), as a TF, could be phosphorylated by Kinectin 1, and these complex could activate the expression of chemokine CXCL8 in invasive basal-like breast cancer (35). Therefore, high-throughput RNA quantification explored the uniqueness of molecular targets for basal-like breast cancer and identified several possible biomarkers for four subtype breast cancer therapeutic strategies.

As known biomarkers for clinical prognosis in breast cancers, such as ER, PR, and HER2, these are essential footstones in layer management, with patients benefiting from endocrine or targeted remedies (36). However, the basal-like subtype still lacks a useable targeted therapy. In our study, we exploited novel biomarkers, E2F8 and MYBL2, which were upregulated specifically in the basal-like subtype. More importantly, high expression of both E2F8 and MYBL2 had a positive correlation with poor patient RFS in the basal-like subtype. Recently, therapy for basal-like patients depends on chemotherapy, so it is an important challenge to explore novel therapeutic drugs, including efficaciously targeted inhibitors and immune-checkpoint inhibitors.

Evidence suggests that both E2F8 and MYBL2 are involved in modulating the cancer cell cycle proteins such as cyclin D1 and cyclin B1 (37, 38). Moreover, all of the hormone ER pathways were repressed by E2F8, and a bit of the hormone ER pathway was activated by MYBL2. It indicated that E2F8 as a basal-like biomarker had a better availability rather than MYBL2. Furthermore, the TIMER database also revealed a positive correlation between E2F8 expression and ICMs in basal-like breast cancer. On the contrary, the MYBL2 expression had no significant correlation with ICMs in the basal-like subtype. Therefore, we considered that patients who had high E2F8 expression were more likely to benefit from immunotherapy than the patients who had high MYBL2 expression. However, PD-L1 protein expression had no significant difference in either siE2F8 or siMYBL2 oligo-treated MDA-MB-231 cells by Western blot assay, but it could not deny the correlation between both E2F8/MYBL2 and PD-L1, because the protein correlation also included phosphorylation regulation, glycosylation, or metabolic activity for these proteins. Interestingly, we also found that the mRNA expression of E2F8 correlated positively with the mRNA expression of MYBL2 in female cancers, including breast cancers (*R* = 0.71; *p* = 3.42e−167), basal-like subtype (*R* = 0.303; *p* = 2.93e−04), ovarian cancers (*R* = 0.652; *p* = 5.17e−37), and cervical squamous cell carcinoma and endocervical adenocarcinoma (*R* = 0.322; *p* = 1.02e−08) (**Supplementary S9**.). If it affects the patients’ immunotherapy, it could be further explored in the future.

Evidence has suggested that tumor-immune interaction in the breast cancer ecosystem is patient-stratifying. Part of the breast cancer patient population is characterized by a number of immune cell infiltrations, including immune-stimulating and immune-inhibiting cells (39). Tumor-associated macrophages and exhausted T cells express PD-L1 at higher levels in both ER+ and ER− tumors (40). While in the basal-like subtype, newly published studies have revealed that a subgroup of patients can benefit from immune-checkpoint inhibitors (41). Blocking PD-L1 has become a necessary means of basal-like breast cancer therapy, enhancing therapeutic efficacy in combination with targeted chemotherapy (42). In our study, we showed that the tumors that had high expression of E2F8 were mostly infiltrated by CD8+ and CD4+ T cells, and that the immune subtype C2 (IFN-gamma dominant) was increased in the tumors with high expression levels of E2F8 or MYBL2.

### CD8+ T cells

Despite having the highest M1 macrophage enrichment and the highest CD8+ T-cell infiltration in in C2 (IFN-γ dominant) subtypes, it showed a poor survival rate for breast cancer patients accompanied by having the higher lymphocytic infiltrate with C2, suggesting complicated regulation by an immune response in the tumor microenvironment (30). Moreover, previous studies have indicated that TFs are viewed as “undruggable” on account of the complexity of active motifs for targeting either DNA–protein or protein–protein interactions (9). Thus, aiming at exploring regulatory pathways for a high level of E2F8 in breast cancers, we discovered predictive inhibitors as follows: PIK-93 and NPK76-II-72-1, which could serve as a therapeutic strategy in patients with high expression of E2F8.

In conclusion, our study showed that E2F8 could be a potential molecular biomarker for basal-like breast cancer. E2F8 deficiency contributed to the suppression of cell migration and invasion in basal-like breast cancer. Furthermore, the knockdown of E2F8 could enhance the sensitivity of docetaxel therapy and inhibit cell proliferation. These findings provided latent molecular targets for basal-like breast cancer treatment.

## Data availability statement

All data that support the findings of this study are available from the corresponding author upon reasonable request. The raw data for RNA-sequence in this article had been deposited in the Genome Sequence Archive (GSA for Human), China National Center for Bioinformation /Beijing Institute of Genomics, Chinese Academy of Sciences (https://ngdc.cncb.ac.cn/gsa-human/s/L06yGb5K: accession no. HRA002753) (43).

## Ethics statement

All surgical samples for breast cancer tissues were approved by the ethics committee of the Shenzhen People’s Hospital (LL-KY-2022384). All the datasets were obtained from the published literature and database online. The informed consents were acquired by the breast cancer patients. The patients/participants provided their written informed consent to participate in this study.

## Author contributions

JZ and JH performed the experiments. JX and LD took charge of analyzing the bioinformatics data. WZ collected breast cancer tissue samples. SL contributed to additional experiments. LG designed this work, analyzed the data, and drafted the manuscript. CZ contributed to the design and supervised this work and revised the manuscript. All authors contributed to the article and approved the submitted version.
