# Supplementary material for: Transcription factor E2F8 is a therapeutic target in the basal-like subtype of breast cancer
Source: Front Oncol. 2023 Feb 6;13:1038787. doi: 10.3389/fonc.2023.1038787 (PMC9939474; doi:10.3389/fonc.2023.1038787)
Supplement: Supplementary file 1 [file DataSheet_1.pdf]

Supplementary Materials for

Transcription factor E2F8 is a therapeutic target in Basal-like subtype of breast cancer

Jing Zheng[1,2#], Jingyi Huang[2#], Jinquan Xia[2,3#], Wenbin Zhou[4], Lingyun Dai[3], Sihang Lin[4], Lin Gao[3*], Chang Zou[3,5,6*]

Chang Zou (zouchang.cuhk@gmail.com); Lin Gao (krystalgao@hotmail.com)

This PDF file includes:

Supplementary Methods

Figures. S1 to S9

Cell Culture and Cell transfection

For cell culture, MDA-MB-231 and BT20 cell lines were purchased from CellCook Biothech CO., LTD. MDA-MB-231 was cultured in DMEM medium (Gibco) contained 10% FBS, and BT20 was cultured in MEM medium (Gibco) supplemnted with 10% FBS and 1% non-essential amino acid (CellCook). All cell lines were incubated at 37°C with 5% CO_2. All cell lines were authenticated using short tandem repeat typing. For cell transfection, cells were treated with siNC oligos, E2F8 small interfering RNA oligos (siRNAs) (siE2F8_1 and siE2F8_2), MYBL2 siRNAs (siMYBL2_1 and siMYBL2_2) (RIBOBIO Biotech CO., Ltd., Guangzhou, China). The cell lines were seeded and cultured to 70%-90% confluence in dish. The mixture contained lipofectamine 3000 (Thermo Fisher Scientific, Waltham, MA, USA) and siRNA oligos was added to the cell lines. The cell lines were incubated for 48 hours at 37°C with 5% CO_2, and total RNA or protein was extracted from cell lysis to analyze. All siRNA oligos sequences as follow in Table S5.

Western blot assay and antibodies

Total protein was isolated from cell lysate by RIPA lysis buffer (Thermo) and performed to analyze as described previously (1). All antibodies as followed: the primary antibodies including anti-E2F8 rabbit pAb (Abclonal, A1135), anti-Cyclin B1 (CCNB1) rabbit pAb (Abclonal, A16800), anti-Cyclin D1 (CCND1) rabbit pAb (Abclonal, A19038), anti-β-Actin rabbit pAb (Abclonal, AC006), and Anti-PD-L1 rabbit pAb (abcam, ab213524). The second antibodies including anti-rabbit IgG- horseradish peroxidase (HRP) (CST, #7074).

Cell Proliferation and Docetaxel Sensitivity Assay

Cell Counting Kit-8 (CCK-8) assay was carried out to assess the docetaxel sensitivity of MDA-MB-231 and BT20 cells. For assessing the IC_{50} value, medium supplemented with docetaxel (DMSO, 0, 0.1, 1, 10, 100, 1000 nM) was performed to culture breast cancer cell lines in 96-well plate for 72 hours. After 72 hours, 10 µL CCK-8 solution (MCE, Monmouth Junction, NJ, USA) was added to the 96-well plate (Corning Inc., Corning, NY, USA) and incubated at 37°C with 5% CO_2. The OD value at 450nm was determined. The cellular IC_{50} value was calculated by GraphPad Prism Software 9.0 (GraphPad Inc, La Jolla, CA, USA). For docetaxel sensitivity assay, MDA-MB-231(8000 per well) or BT20 cells (6000 per well) cultivated on 96-well plate were treated with siRNA oligos and/or docetaxel, and cell

proliferation was determined after 72 h using a Cell Counting Kit 8 (CCK-8), with the results being recorded at 450 nm according to the manufacturers' instructions.

Cell Migration and Invasion

Briefly, for the cell migration assay, 1 × 10^5 breast cancer cells-treated with E2F8 or MYBL2 siRNA oligos were seeded in Transwell chambers (Merck Millipore, Billerica, MA, USA; 8 μm) in 24-well plate (Corning Inc.). After being cultured for 20 hours, the migrated cells were fixed by 4% paraformaldehyde, and stained by 0.1% crystal violet, and counted. Each experiment was repeated at least three times. For the cell invasion assay, 1 × 10^5 cells were seeded in Matrigel-coated Transwell chambers (BD, 8 μm) in 24-well plate. Other procedures were the same as the cell migration assay.

Table S1. All differentially expressed TFs between Luminal A Ca and P [|log2(fold change) |>3)]

Gene_ID	Gene name	log_2(fold change)	*P*-value	Up/Down
ENSG00000159184	HOXB13	9.560511	0.001584	Up
ENSG00000144331	ZNF385B	5.312519	2.26E-05	Up
ENSG00000101057	MYBL2	5.069454	0.000333	Up
ENSG00000069011	PITX1	3.727384	0.00338	Up
ENSG00000253831	ETV3L	-7.18466	2.00E-05	Down
ENSG00000174963	ZIC4	-7.20117	0.020371	Down
ENSG00000009950	MLXIPL	-4.07976	0.00219	Down
ENSG00000100146	SOX10	-3.94128	0.002708	Down
ENSG00000005513	SOX8	-3.38829	0.01314	Down
ENSG00000168874	ATOH8	-3.43519	7.94E-05	Down
ENSG00000170549	IRX1	-3.18432	0.017861	Down
ENSG00000135374	ELF5	-4.94334	2.22E-05	Down
ENSG00000108924	HLF	-3.37499	1.27E-12	Down

Table S2. All differentially expressed TFs between Luminal B Ca and P [|log2(fold change) |>5)]

Gene_ID	Gene name	log_2(fold change)	*P*-value	Up/Down
ENSG00000182111	ZNF716	22.44177	9.30E-09	Up
ENSG00000159184	HOXB13	9.072158	0.004173	Up
ENSG00000136574	GATA4	21.03914	7.36E-08	Up
ENSG00000123407	HOXC12	5.784067	0.000785	Up
ENSG00000173404	INSM1	8.962367	3.26E-07	Up
ENSG00000171872	KLF17	9.37605	0.000903	Up
ENSG00000196132	MYT1	7.513141	1.81E-05	Up

ENSG00000198597	ZNF536	-11.2509	2.79E-08	Down
ENSG00000253831	ETV3L	-9.14005	2.97E-07	Down
ENSG00000198914	POU3F3	-6.55327	0.000615	Down
ENSG00000009950	MLXIPL	-5.40059	1.62E-08	Down
ENSG00000100146	SOX10	-8.40297	4.33E-26	Down
ENSG00000159387	IRX6	-6.04947	2.59E-09	Down
ENSG00000143867	OSR1	-5.93868	9.29E-22	Down
ENSG00000005102	MEOX1	-5.01674	5.57E-12	Down
ENSG00000163884	KLF15	-6.15163	1.07E-34	Down
ENSG00000168874	ATOH8	-6.49349	3.75E-19	Down
ENSG00000170549	IRX1	-5.06473	1.02E-10	Down
ENSG00000108924	HLF	-5.73671	4.83E-18	Down
ENSG00000185002	RFX6	-8.54886	0.000267	Down
ENSG00000185610	DBX2	-7.21919	2.97E-09	Down
ENSG00000180318	ALX1	-23.2164	2.83E-09	Down
ENSG00000124440	HIF3A	-6.32676	1.19E-07	Down
ENSG00000183733	FIGLA	-9.21136	7.06E-06	Down
ENSG00000185960	SHOX	-5.21573	0.008424	Down

Table S3. All differentially expressed TFs between HER2 Ca and P [|log2(fold change) |>5)]

Gene_ID	Gene name	$\log_2$(fold change)	*P*-value	Up/Down
ENSG00000131914	LIN28A	11.56129	0.003084	Up
ENSG00000182111	ZNF716	9.790643	0.000464	Up
ENSG00000123388	HOXC11	5.917815	1.38E-12	Up
ENSG00000249961	TERB1	8.871411	0.003347	Up
ENSG00000091010	POU4F3	5.748436	0.006244	Up
ENSG00000111783	RFX4	25.59867	5.67E-11	Up
ENSG00000169856	ONECUT1	7.516253	0.000258	Up
ENSG00000142025	DMRTC2	8.480423	0.000379	Up
ENSG00000159184	HOXB13	7.851638	2.10E-05	Up
ENSG00000176887	SOX11	5.331114	5.06E-07	Up
ENSG00000123407	HOXC12	6.652723	0.00023	Up
ENSG00000130675	MNX1	5.889985	1.31E-06	Up
ENSG00000123364	HOXC13	5.77137	8.39E-07	Up
ENSG00000125820	NKX2-2	11.00127	0.004866	Up
ENSG00000159263	SIM2	5.298657	7.00E-11	Up
ENSG00000069011	PITX1	6.592349	2.48E-39	Up
ENSG00000009709	PAX7	26.93122	5.44E-12	Up
ENSG00000119547	ONECUT2	6.746051	6.56E-08	Up
ENSG00000175329	ISX	20.81307	1.02E-07	Up
ENSG00000162624	LHX8	26.73771	7.71E-12	Up
ENSG00000198597	ZNF536	-9.37792	1.76E-06	Down
ENSG00000182348	ZNF804B	-12.7572	0.001093	Down
ENSG00000052850	ALX4	-6.95208	7.65E-16	Down
ENSG00000253831	ETV3L	-5.72724	7.47E-07	Down

ENSG00000100146	SOX10	-8.52036	6.67E-46	Down
ENSG00000143867	OSR1	-7.3358	6.67E-28	Down
ENSG00000212993	POU5F1B	-8.75083	0.004671	Down
ENSG00000170549	IRX1	-5.86871	3.12E-24	Down
ENSG00000163792	TCF23	-6.63369	0.000422	Down
ENSG00000162761	LMX1A	-5.62664	0.001085	Down
ENSG00000109906	ZBTB16	-5.33832	3.41E-08	Down
ENSG00000180318	ALX1	-21.592	3.34E-08	Down

Table S4. All differentially expressed TFs between Basal Ca and P [| $\log_2$(fold change) |>5)]

Gene_ID	Gene name	$\log_2$(fold change)	*P*-value	Up/Down
ENSG00000164438	TLX3	8.612965	4.00E-05	Up
ENSG00000187772	LIN28B	10.6527	3.40E-05	Up
ENSG00000130711	PRDM12	7.474135	0.003214	Up
ENSG00000142700	DMRTA2	8.349861	0.000684	Up
ENSG00000107807	TLX1	8.891876	5.85E-10	Up
ENSG00000164256	PRDM9	20.99933	7.80E-08	Up
ENSG00000007372	PAX6	5.394424	2.37E-05	Up
ENSG00000184937	WT1	5.378228	4.88E-06	Up
ENSG00000148704	VAX1	23.58816	9.92E-17	Up
ENSG00000184486	POU3F2	7.183708	9.56E-05	Up
ENSG00000159184	HOXB13	7.781013	9.22E-05	Up
ENSG00000138083	SIX3	6.031319	6.66E-10	Up
ENSG00000137090	DMRT1	11.91024	0.0023	Up
ENSG00000176887	SOX11	7.086752	1.17E-16	Up
ENSG00000136574	GATA4	8.770774	0.003424	Up
ENSG00000152977	ZIC1	5.634499	8.66E-11	Up
ENSG00000183072	NKX2-5	5.506988	3.32E-06	Up
ENSG00000269404	SPIB	5.681352	6.00E-07	Up
ENSG00000101057	MYBL2	5.722797	2.53E-30	Up
ENSG00000159263	SIM2	7.420735	1.16E-31	Up
ENSG00000129173	E2F8	5.482486	1.14E-24	Up
ENSG00000256463	SALL3	7.278178	0.000338	Up
ENSG00000123576	ESX1	22.24699	1.25E-08	Up
ENSG00000196092	PAX5	5.082448	3.74E-15	Up
ENSG00000197472	ZNF695	6.417163	2.50E-47	Up
ENSG00000106689	LHX2	8.515652	1.10E-05	Up
ENSG00000215397	SCRT2	20.45251	1.67E-07	Up
ENSG00000124092	CTCFL	22.59964	2.25E-09	Up
ENSG00000182348	ZNF804B	-11.5645	1.36E-14	Down
ENSG00000186766	FOXI2	-5.2363	0.028124	Down
ENSG00000139445	FOXN4	-23.1728	1.56E-09	Down
ENSG00000185002	RFX6	-6.94654	0.000339	Down
ENSG00000162761	LMX1A	-7.97486	0.0051	Down

Table S5. Sequences of qRT-PCR primers used in this study.

	Primers
**MYBL2**	Forward: 5’-TGTGGATGAGGATGTGAAGC-3’
	Reverse: 5’-TGAGGCTGGAAGAGTTTGAAG-3’
**HOXC13**	Forward: 5’-GTCAGGTGTACTGCTCCAAG-3’
	Reverse: 5’-CTCCTTCAGCTGCACCTTAG-3’
**E2F8**	Forward: 5’-TGACATCTGCCTTGACGAAG-3’
	Reverse: 5’-TGTTGAGATTGTGTCGCCC-3’

Table S6. Sequences of siRNA oligos used in this study.

	Sequences
**siMYBL2_1**	5’-CCTGTCAGGTATCAAAGAA-3’
**siMYBL2_2**	5’-GGAAGTCTCTGGCTCTTGA-3’
**siE2F8_1**	5’-CGACACCCATCTCTTATCA-3’
**siE2F8_2**	5’-GCTCGCAGCTATTTGTAAA-3’

[1] L. Gao, W. Zhou, N. Xie, J. Qiu, J. Huang, Z. Zhang, et al. Yin Yang 1 promotes aggressive cell growth in high-grade breast cancer by directly transactivating kinectin 1. MedComm (2020) 2022;3:e133.

Supplementary Figure. S1

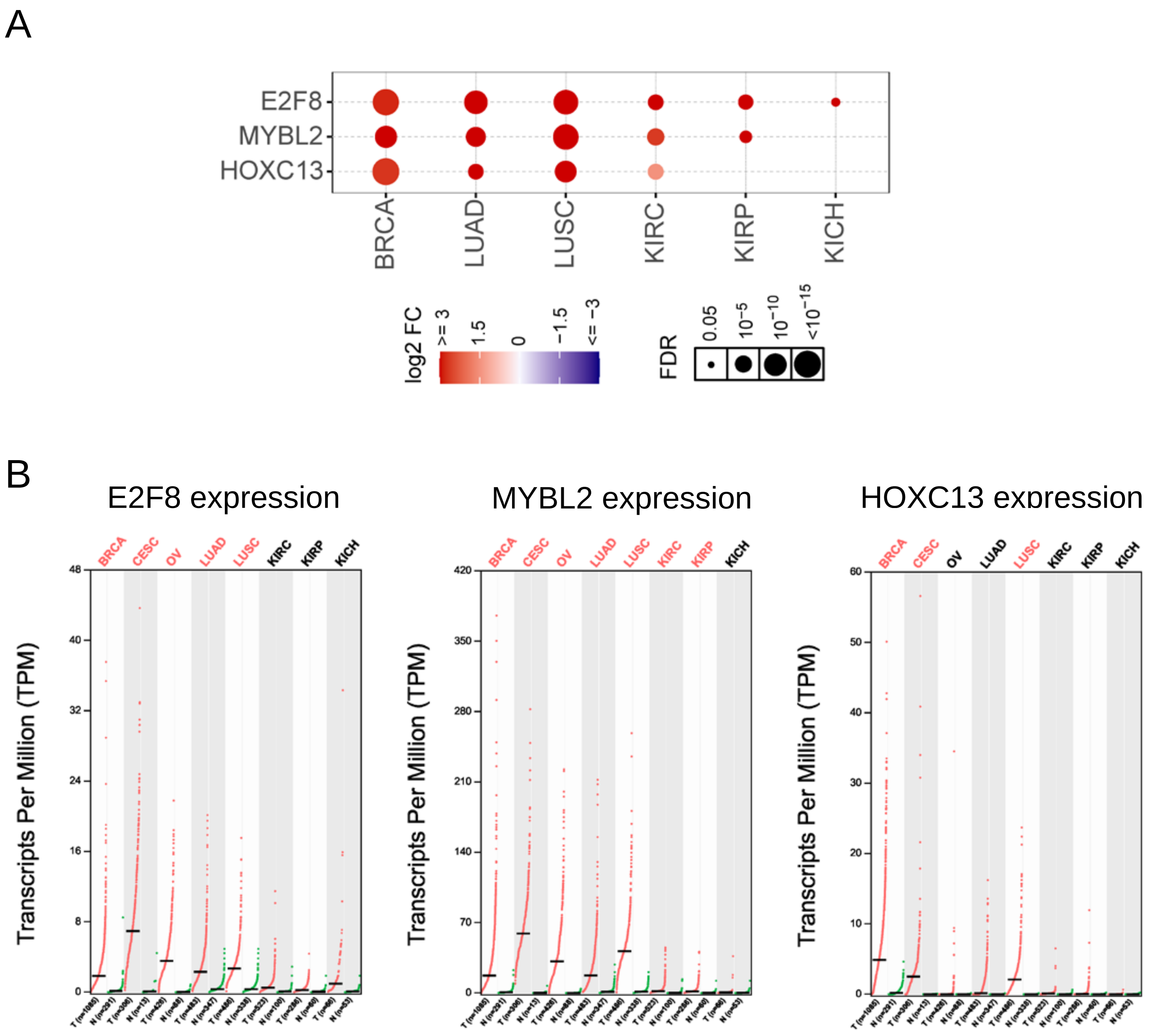

Supplementary Figure S1 （A）The differential expression levels of these TFs in pan-cancer from GSCA Lite database. （B）The differential expression levels of these TFs in pan-cancer from GEPIA database.

Supplementary Figure. S2

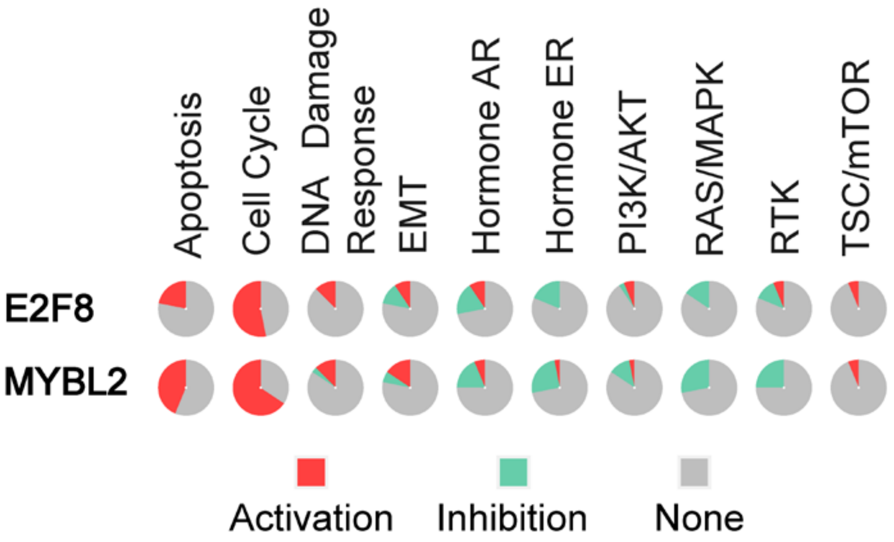

Supplementary Figure S2 The signalling pathway analysis of having been screened TFs using the GSCALite online tool.

Supplementary Figure. S3

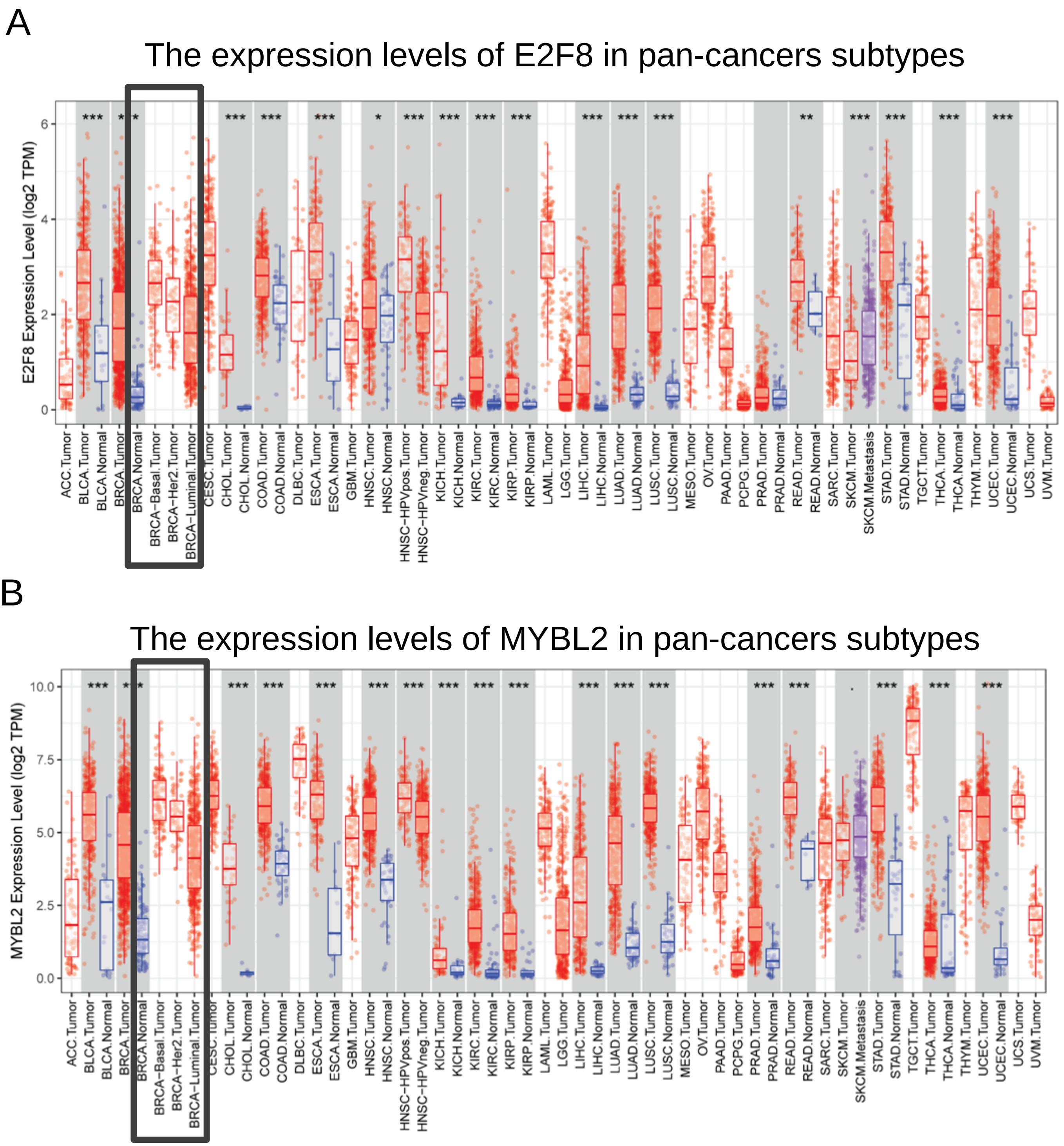

Supplementary Figure S3 (**A**) The expression levels of E2F8 in pan-cancers subtypes compared with normal tissues using TIMER dataset online. (**B**) The expression levels of MYBL2 in pan-cancers subtypes compared with normal tissues using TIMER dataset online.

Supplementary Figure. S4

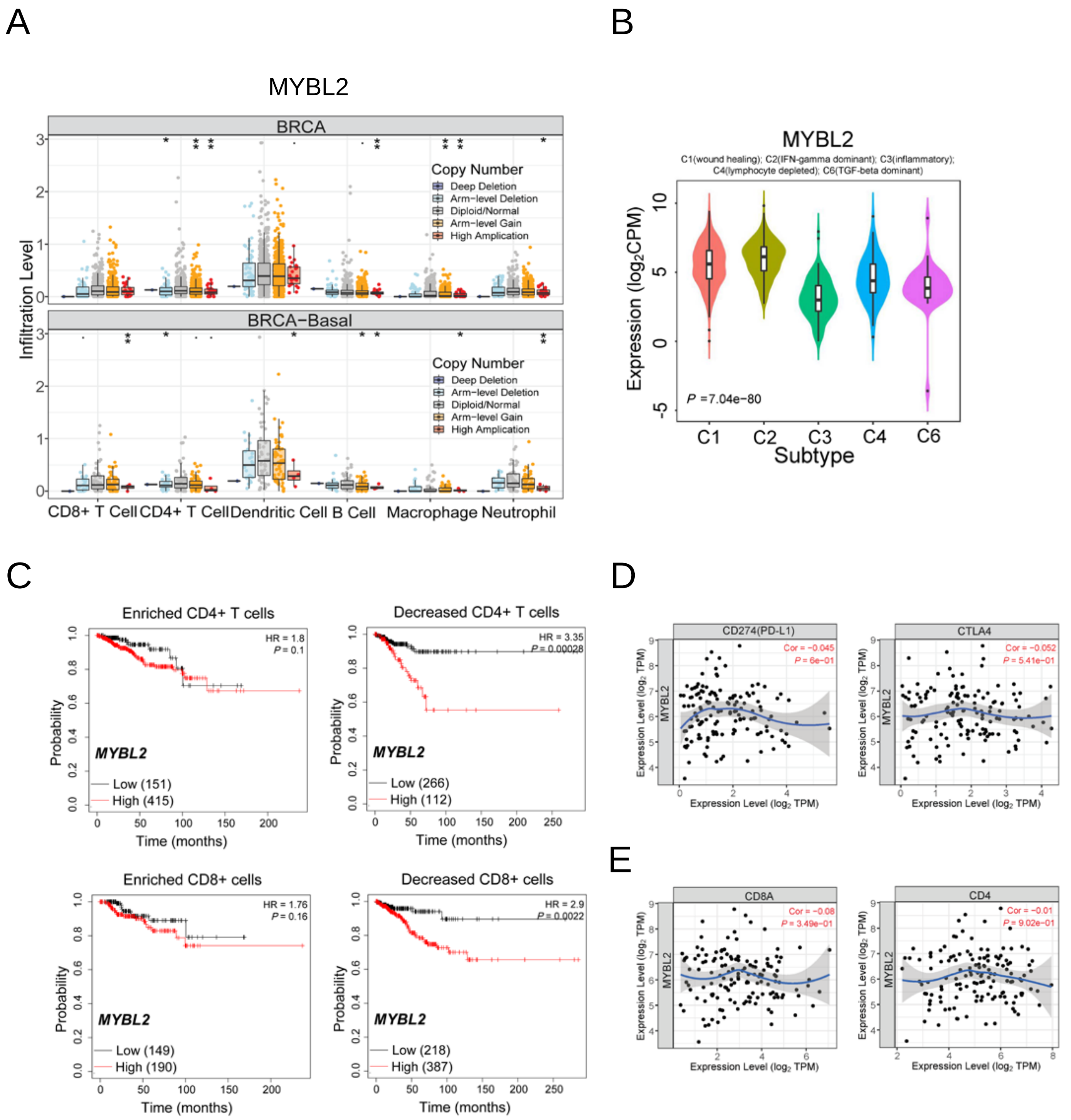

Supplementary Figure S4 (A) The immune cellular infiltrates analysis in high expression level of MYBL2 in Basal-like subtypes and BRCA by TISIDB database. **(B)** The correlation between MYBL2 expression and immune subtypes analysis in invasive breast cancers. Classic classification as follow: C1 (wound healing); C2 (IFN-γ dominant); C3 (inflammatory); C4 (lymphocyte depleted); C5 (immunologically quiet); C6 (TGF-β dominant). **(C)** The overall survival (OS) analysis of breast cancer patients by the Kaplan-Meier plotter dataset. The OS groupings covered enriched or decreased macrophage cells in the patients who had ‘high’ or ‘low’ expression of MYBL2. **(D)** The correlation between MYBL2 expression and immune checkpoint gene (PD-L1 and CTLA4). **(E)** The correlation between MYBL2 expression and CD8A/CD4.

Supplementary Figure. S5

A

The correlation between E2F8 expression and immune cell infiltration in breast cancers

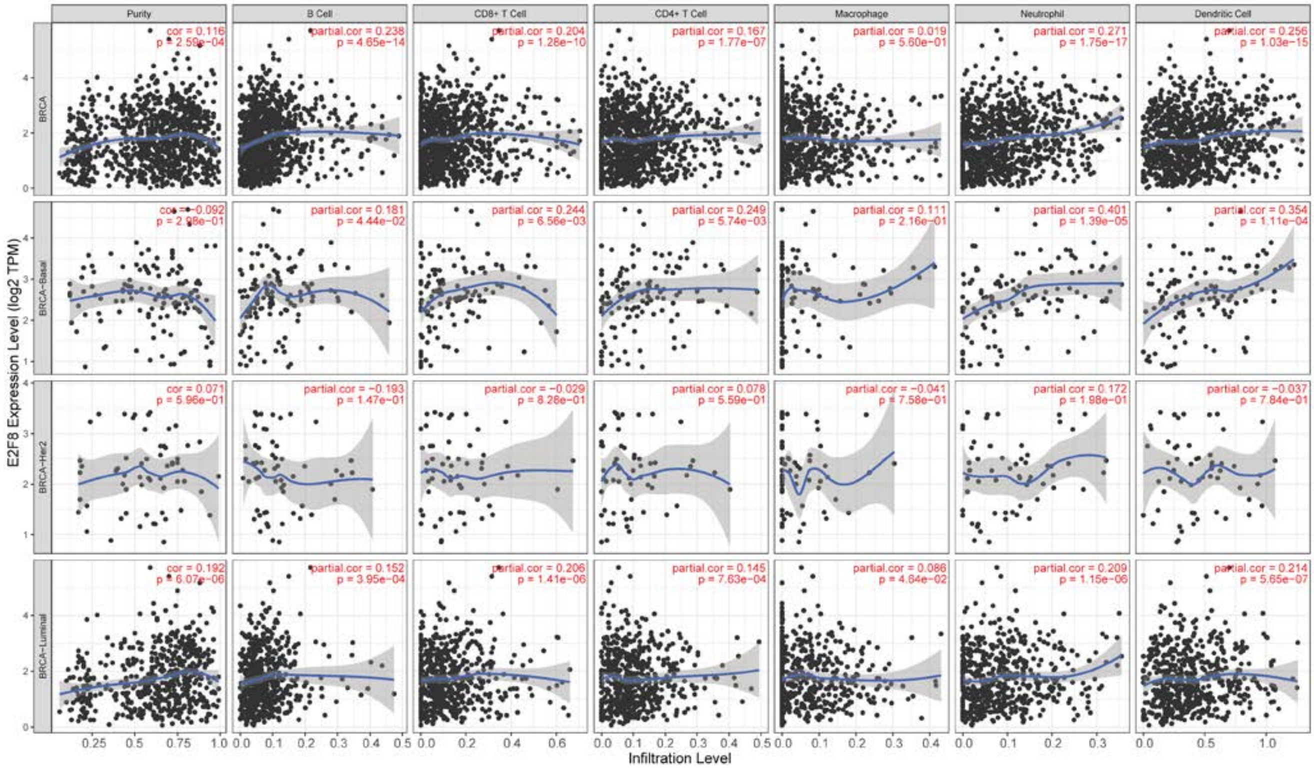

B

The correlation between MYBL2 expression and immune cell infiltration in breast cancers

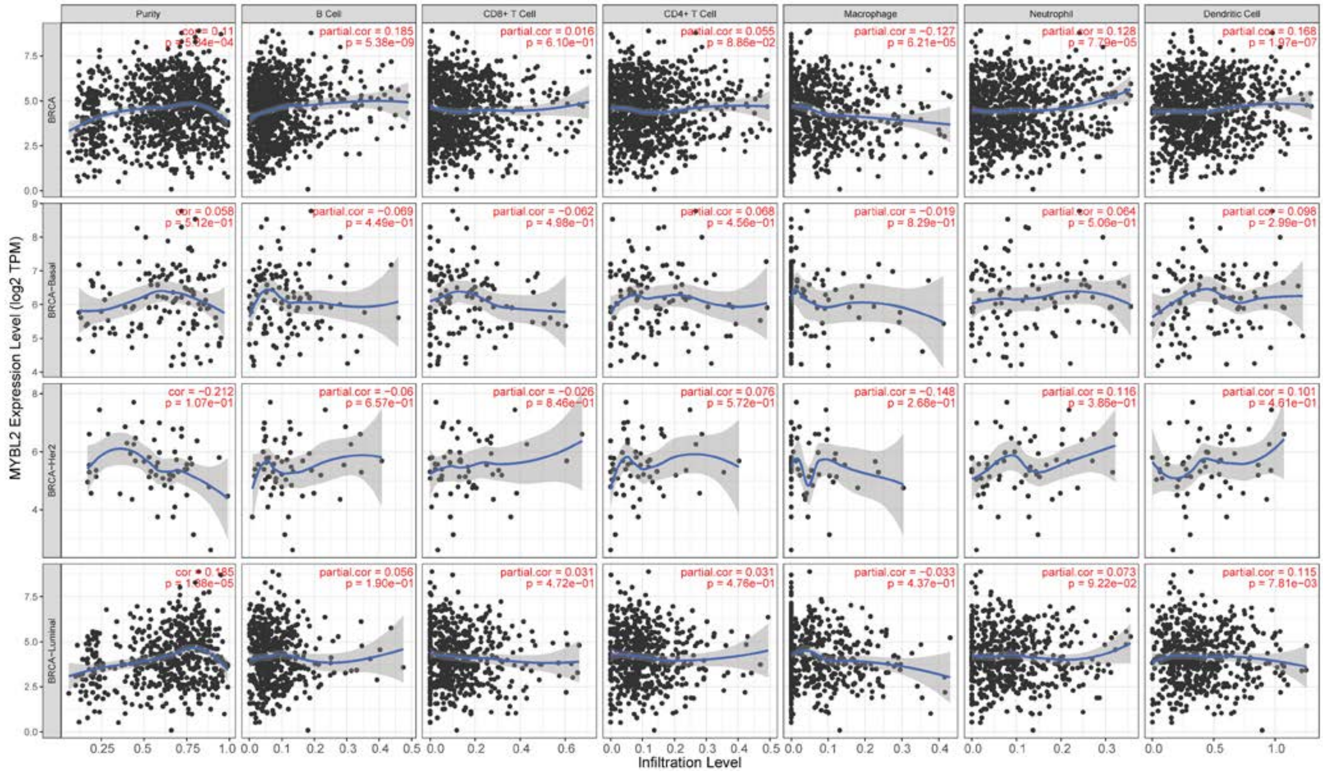

Supplementary Figure S5 The correlation between E2F8 (/MYBL2) expression and immune cell infiltration in breast cancers by TIMER database.

Supplementary Figure. S6

A

The correlation between E2F8 and immune checkpoint molecules (ICMs) in Basal-like breast cancer

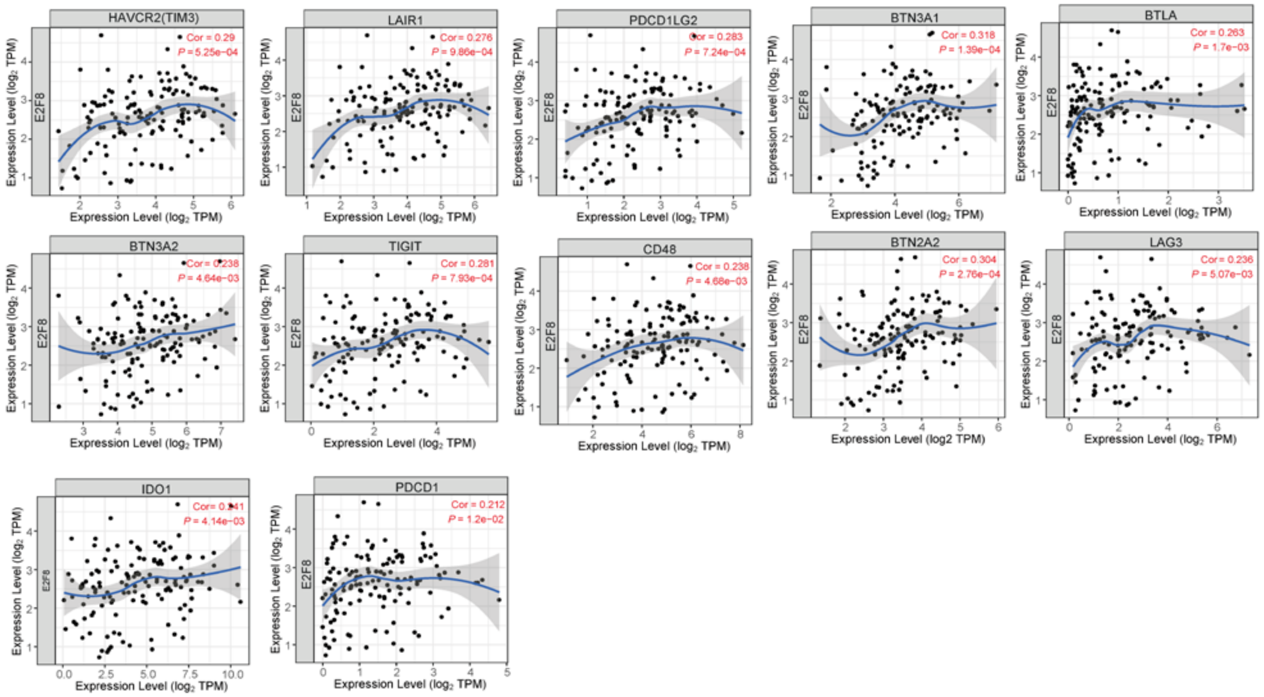

B

The correlation between MYBL2 and immune checkpoint molecules (ICMs) in Basal-like breast cancer

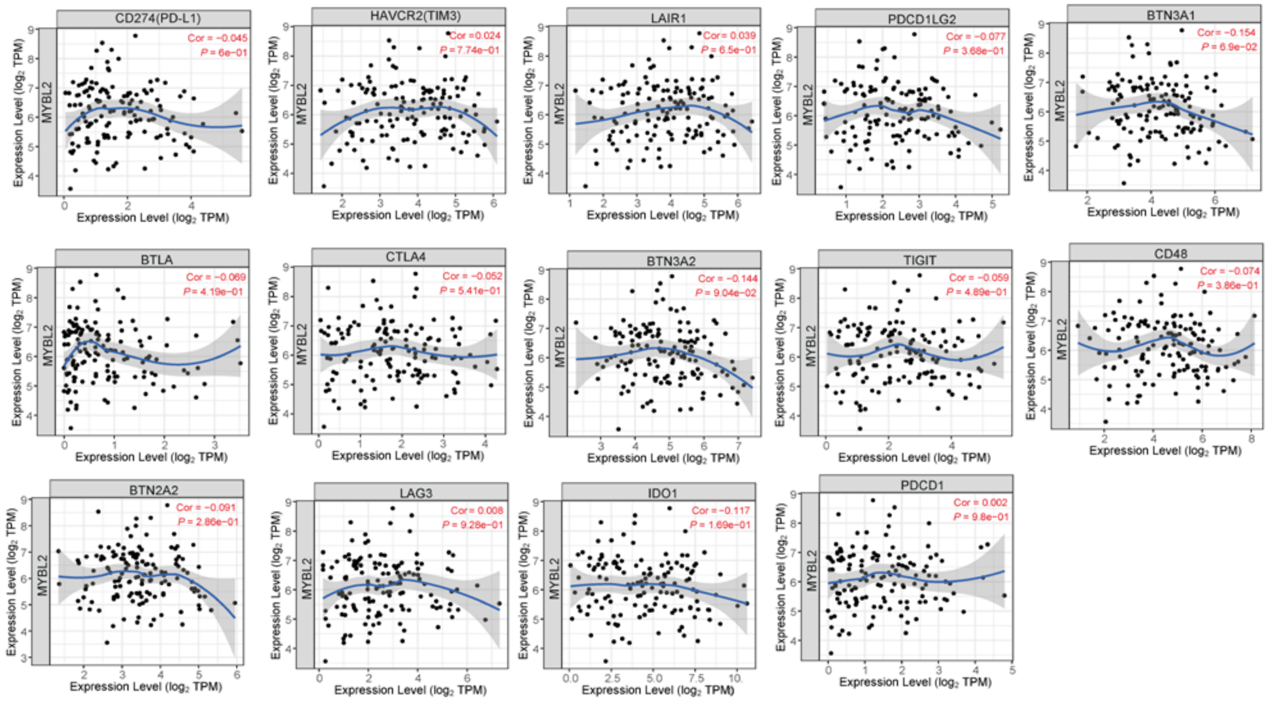

Supplementary Figure S6 The correlation between E2F8 (/MYBL2) and immune checkpoint molecules (ICMs) in Basal-like breast cancer by TIMER database. **(A)** E2F8; **(B)** MYBL2.

Supplementary Figure. S7

A

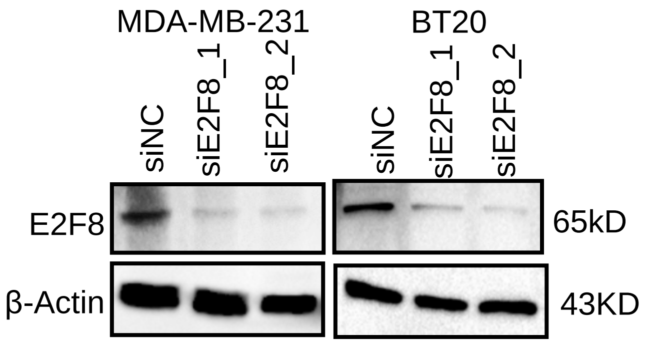

B

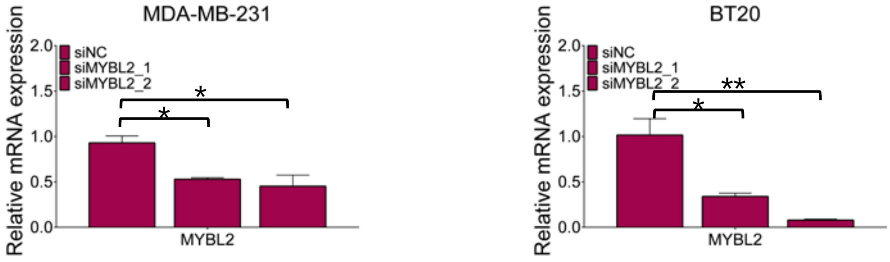

Supplementary Figure S7 (A) The expression of E2F8 in MDA-MB-231 and BT20 cells treated with E2F8 siRNA compared with siNC as assessed by western blot assay. **(B)** Knockdown of MYBL2 in MDA-MB-231 and BT20 cells treated with MYBL2 siRNA compared with siNC as assessed by qRT-PCR. **P*<0.05, ***P*<0.01 and ****P*<0.001 compared with siNC group.

Supplementary Figure. S8

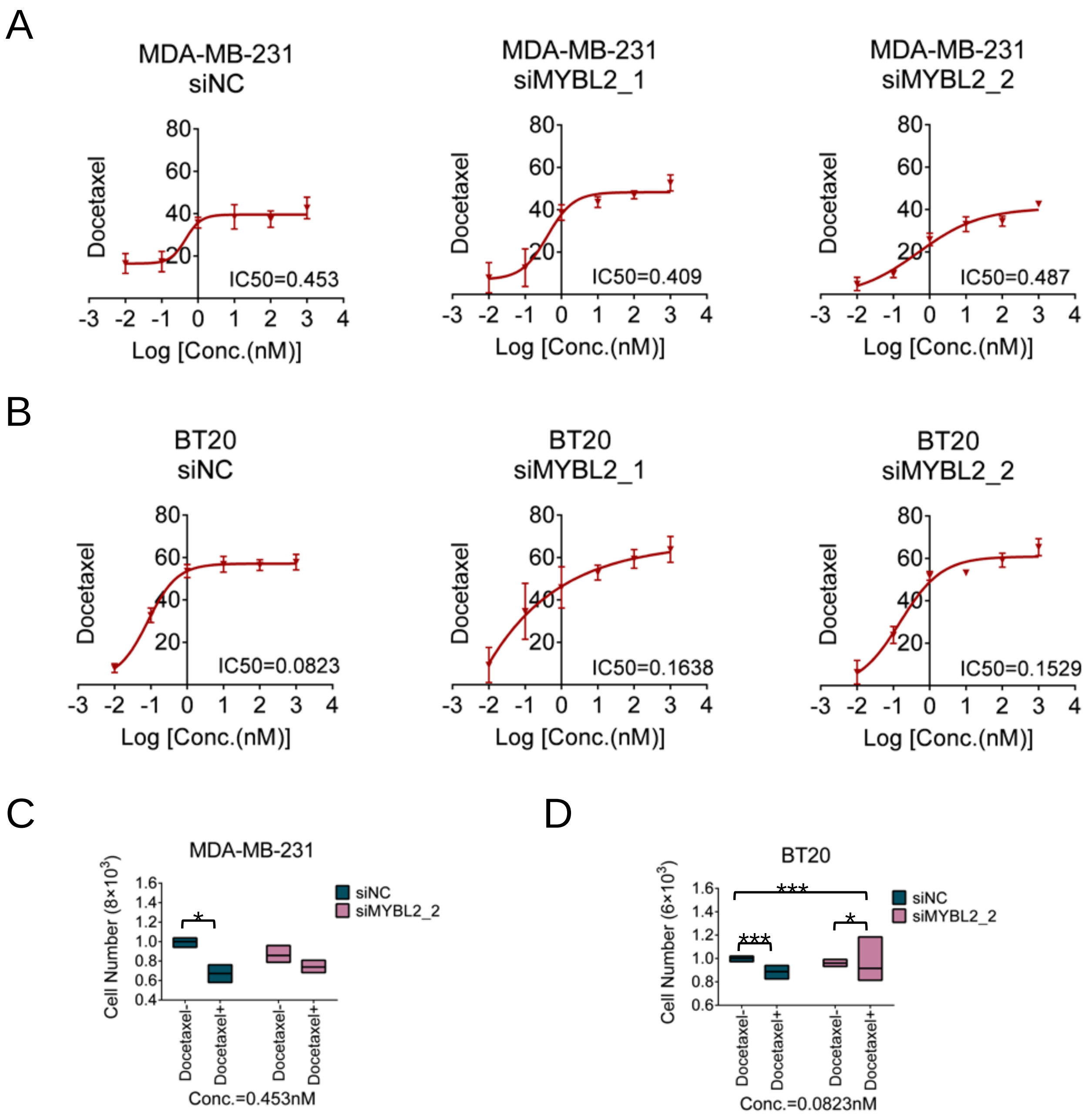

Supplementary Figure S8 CCK-8 analysis of the IC50 of cell treated with docetaxel for siNC group, siE2F8_1 group, and siE2F8_2 group in **(A)** MDA-MB-231 cell and **(B)** BT20 cell lines. CCK-8 analysis of the drug susceptibility for siNC group, siNC with docetaxel-treated group, siE2F8 group, and siE2F8 with docetaxel-treated group in **(C)** MDA-MB-231 and **(D)** BT20 cell lines.

Supplementary Figure. S9

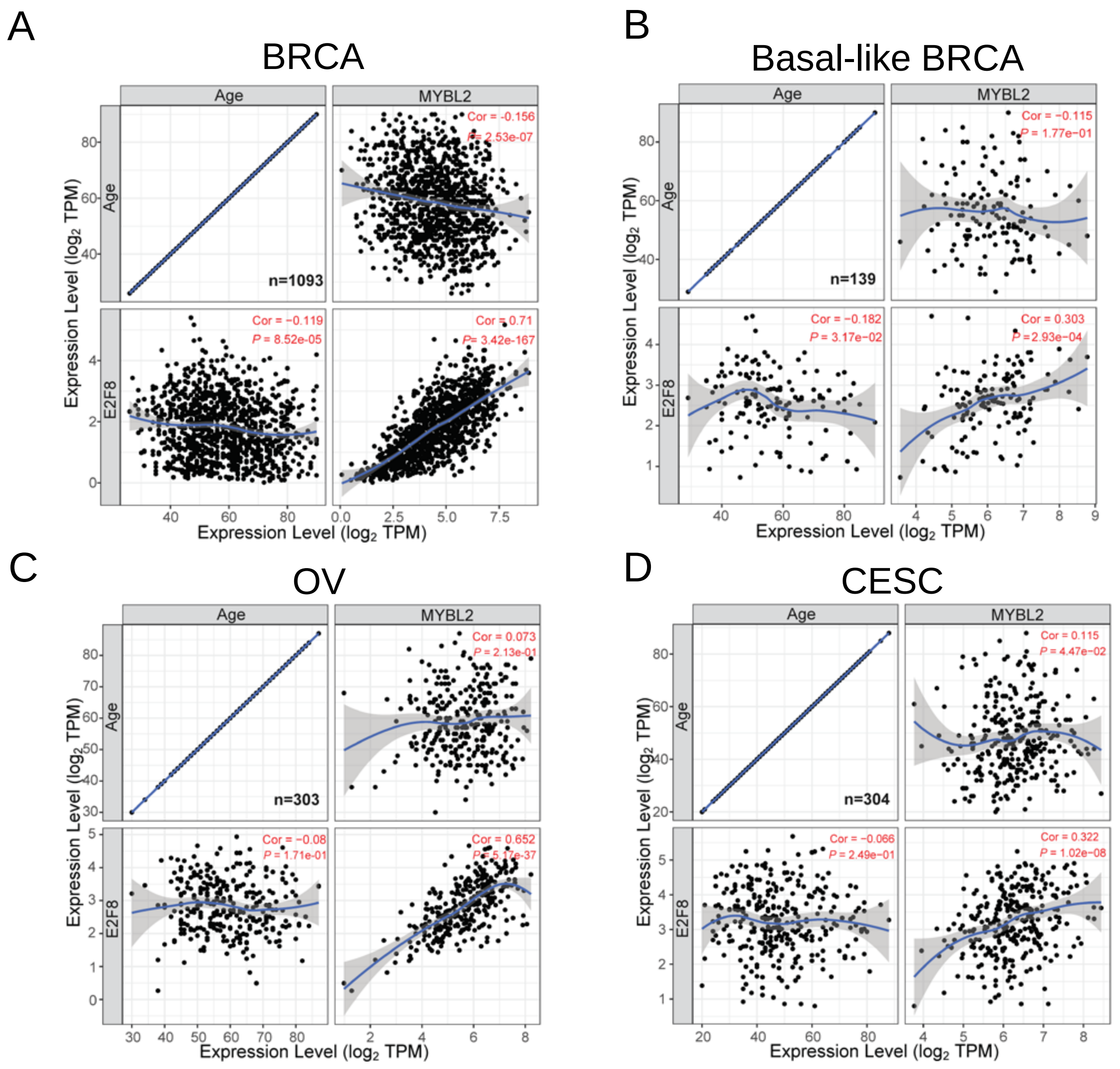

Supplementary Figure S9 The correlation between E2F8 and MYBL2 expression in female tumors including (**A**) breast cancers (BRCA), (**B**) Basal-like subtypes (Basal-like BRCA), (**C**) ovarian cancer (OV), and (**D**) Cervical squamous cell carcinoma and endocervical adenocarcinoma (CESC) by TIMER database.
